# Quantitative evaluation of PSMA PET imaging using a realistic anthropomorphic phantom and shell-less radioactive epoxy lesions

**DOI:** 10.1186/s40658-021-00429-9

**Published:** 2022-01-15

**Authors:** Roberto Fedrigo, Dan J. Kadrmas, Patricia E. Edem, Lauren Fougner, Ivan S. Klyuzhin, M. Peter Petric, François Bénard, Arman Rahmim, Carlos Uribe

**Affiliations:** 1 Department of Integrative Oncology, BC Cancer Research Institute, 675 W 10th Avenue, Vancouver, BC V5Z1L3 Canada; 2 Department of Physics and Astronomy, University of British Columbia, 325-6224 Agricultural Road, Vancouver, BC V6T1Z1 Canada; 3 Department of Radiology and Imaging Sciences, University of Utah, 201 Presidents’ Cir, Salt Lake City, UT 84112 USA; 4 Functional Imaging, BC Cancer, 600 W 10th Avenue, Vancouver, BC V5Z4E6 Canada; 5 Department of Molecular Oncology, BC Cancer Research Institute, 675 W 10th Avenue, Vancouver, BC V5Z1L3 Canada; 6 Department of Radiology, University of British Columbia, 675 W 10th Avenue, Vancouver, BC V5Z1L3 Canada

**Keywords:** PSMA, Phantoms, Segmentation, PET

## Abstract

**Background:**

Positron emission tomography (PET) with prostate specific membrane antigen (PSMA) have shown superior performance in detecting metastatic prostate cancers. Relative to [^18^F]fluorodeoxyglucose ([^18^F]FDG) PET images, PSMA PET images tend to visualize significantly higher-contrast focal lesions. We aim to evaluate segmentation and reconstruction algorithms in this emerging context. Specifically, Bayesian or maximum a posteriori (MAP) image reconstruction, compared to standard ordered subsets expectation maximization (OSEM) reconstruction, has received significant interest for its potential to reach convergence with minimal noise amplifications. However, few phantom studies have evaluated the quantitative accuracy of such reconstructions for high contrast, small lesions (sub-10 mm) that are typically observed in PSMA images. In this study, we cast 3 mm–16-mm spheres using epoxy resin infused with a long half-life positron emitter (sodium-22; ^22^Na) to simulate prostate cancer metastasis. The anthropomorphic Probe-IQ phantom, which features a liver, bladder, lungs, and ureters, was used to model relevant anatomy. Dynamic PET acquisitions were acquired and images were reconstructed with OSEM (varying subsets and iterations) and BSREM (varying *β* parameters), and the effects on lesion quantitation were evaluated.

**Results:**

The ^22^Na lesions were scanned against an aqueous solution containing fluorine-18 (^18^F) as the background. Regions-of-interest were drawn with MIM Software using 40% fixed threshold (40% FT) and a gradient segmentation algorithm (MIM’s PET Edge^+^). Recovery coefficients (RCs) (max, mean, peak, and newly defined “apex”), metabolic tumour volume (MTV), and total tumour uptake (TTU) were calculated for each sphere. SUV_peak_ and SUV_apex_ had the most consistent RCs for different lesion-to-background ratios and reconstruction parameters. The gradient-based segmentation algorithm was more accurate than 40% FT for determining MTV and TTU, particularly for lesions $\le$ 6 mm in diameter (*R*^2^ = 0.979–0.996 vs. *R*^2^ = 0.115–0.527, respectively).

**Conclusion:**

An anthropomorphic phantom was used to evaluate quantitation for PSMA PET imaging of metastatic prostate cancer lesions. BSREM with *β* = 200–400 and OSEM with 2–5 iterations resulted in the most accurate and robust measurements of SUV_mean_, MTV, and TTU for imaging conditions in ^18^F-PSMA PET/CT images. SUV_apex_, a hybrid metric of SUV_max_ and SUV_peak_, was proposed for robust, accurate, and segmentation-free quantitation of lesions for PSMA PET.

**Supplementary Information:**

The online version contains supplementary material available at 10.1186/s40658-021-00429-9.

## Introduction

Prostate cancer (PCa) is the second most prevalent malignancy in men and fifth deadliest worldwide [1]. Although locally contained prostate cancer has high (> 90%) survival, metastatic prostate cancer (mPCa) only has 30% 5-year survival rates [2]. A new generation of pharmaceuticals that target the prostate specific membrane antigen (PSMA) has shown high specificity for detecting mPCa. Various PSMA ligands have been developed for applications to positron emission tomography (PET), such as using gallium-68 (^68^Ga) or fluorine-18 (^18^F) radioisotopes [3]. For instance, PET images with the recently approved (US Food and Drug Administration) PSMA-targeting tracer, [^18^F]DCFPyL, have shown superior results in detecting mPCa [4, 5] allowing for observation of high contrast and focal lesions. Moreover, [^18^F]DCFPyL PET has improved treatment decisions and patient management of PCa patients [4].

Accurate quantitation of lesions imaged with PSMA PET can enable improved evaluation of therapeutic efficacy, harmonization between sites, and the potential to build outcome predictive models. Conventionally, scanner performance has been validated using the NEMA Image Quality phantom, in which ^18^F is injected into 10–37-mm fillable spheres at 4:1 lesion-to-background activity ratios [6, 7]. The NEMA approach, however, does not approximate the high-contrast, low-diameter lesions that are characteristics of PCa imaging with PSMA-based agents. Furthermore, the NEMA approach can create “cold shell” artefacts around the spheres because the plastic walls displace background activity. The relative volume of a “cold shell” becomes significant for small diameter lesions, and has been found to reduce the measured concentration [8] and increase the observed volume of a lesion [9]. Alternate methods have been developed to circumvent the cold shell effect, such as by casting radioactive spheres [8, 10, 11] or inserting spheres into the background using superposition [12, 13], but few studies have evaluated quantitation for focal, high-contrast lesions. Additionally, conventional phantom experiments do not realistically model organs that are highly relevant to PSMA imaging. For instance, PSMA patients characteristically exhibit high liver and bladder uptake [14, 15], which can greatly influence detection and quantitation of nearby tumours. Anthropomorphic phantoms, such as the Probe-IQ phantom [10, 16–20], represent a significant advancement within phantom technology. The Probe-IQ phantom simulates realistic thorax and pelvic regions, while also accounting for major organs such as the liver, bladder, and lungs. Furthermore, Probe-IQ utilizes unique filter foam technology to establish heterogeneous radioactivity distributions within the phantom compartments, making it more representative of a human PET scan [10, 20].

PET scans are commonly reconstructed using the ordered subsets expectation maximization (OSEM) algorithm. A limitation of this algorithm is that noise is amplified at high number of iterations, so limited iterations are used in practice to maintain adequate image quality—often at the expense of radioactivity quantification accuracy resulting from lower standardized uptake values (SUV) [21]. Block sequential regularized expectation maximization (BSREM) is a penalized-likelihood reconstruction algorithm that has received significant interest for its potential to reach convergence with minimal noise amplifications [22, 23].

This study aimed at evaluating mPCa within the emerging context of PSMA PET imaging. Rather than performing this task with a standardized phantom, we insert “shell-less” radioactive spheres into the highly realistic Probe-IQ phantom to model prostate cancer metastasis. Tumour radioactivity concentration, volume, and uptake were evaluated across a range of OSEM and BSREM reconstruction parameters and lesion-to-background ratios, characterizing the accuracy and robustness of each segmentation method. Lastly, we consider the trade-off between image quality and quantitation to identify reconstruction parameters and segmentation methods that may be most appropriate for PSMA PET imaging.

## Methods

### Anthropomorphic phantom

**Fig. 1:**
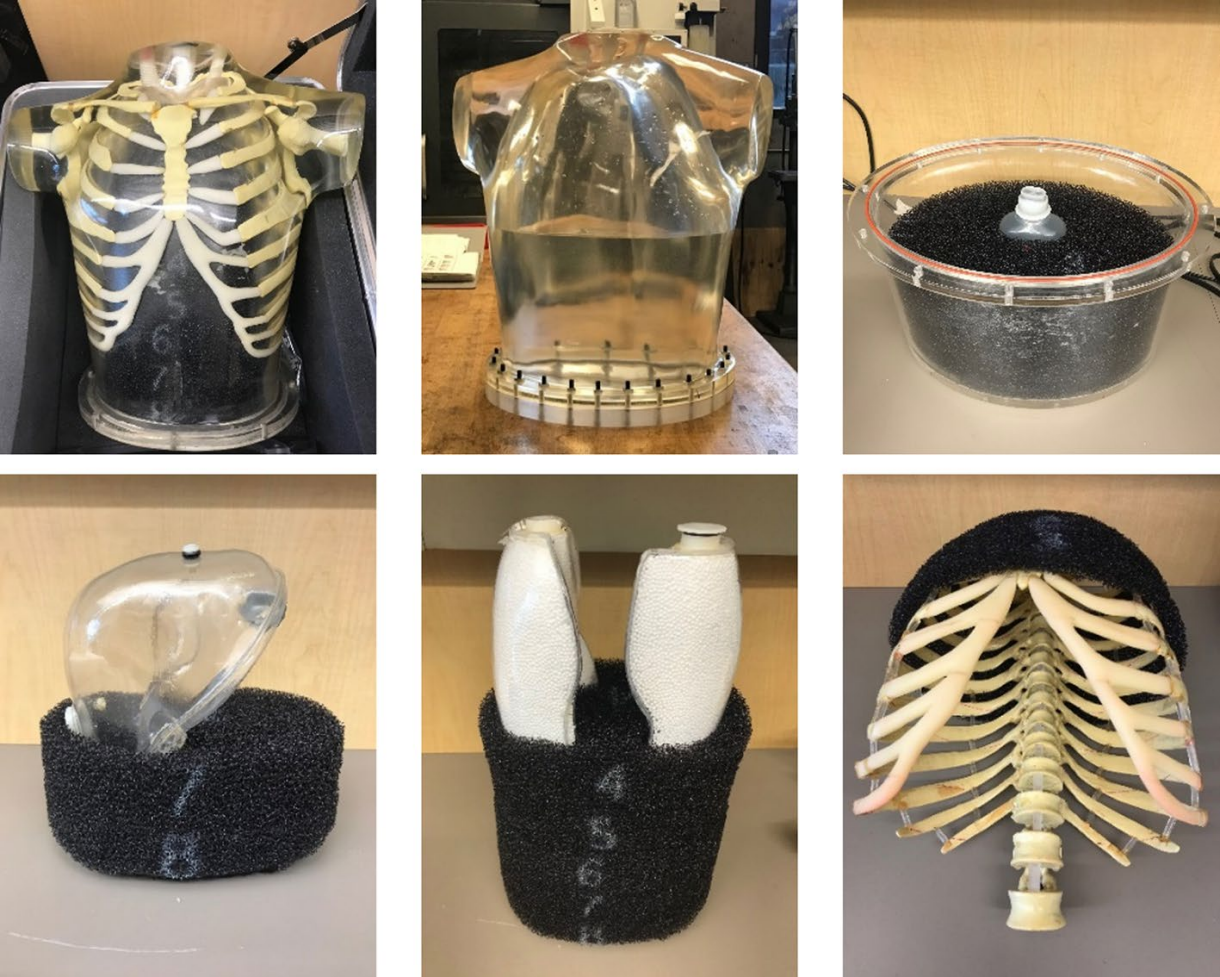
Anthropomorphic Probe-IQ Phantom. (Top) Left to Right: medium phantom shell, large phantom shell, pelvis shell with bladder insert. (Bottom) Left to Right: liver, lung inserts, ribs and spine that can be inserted in the large phantom

The highly realistic Probe-IQ phantom (Fig. 1) consists of a thorax (Radiology Support Devices, Inc., USA) and pelvis phantom (Data Spectrum Corp., USA). The thorax contains a fillable liver, lungs, ribs, and spinal cord, and was designed to simulate the size of a 92-kg patient [18]. Nylon mesh bags containing Styrofoam beads (Dow Chemical Co., Midland, MI, USA) were placed inside the lung inserts to lower the activity concentration to 37% of the background concentration [20]. The beads also simulated realistic lung tissue density [20, 24]. The Probe-IQ pelvis contains a 440-mL compartment and clinical-grade tubing to simulate the bladder and ureters, respectively. Polyurethane filter foam was used to position the organs within the phantom and create small pockets of air bubbles within the foam, to establish heterogeneous background activity which is more representative of a real human scan [10, 20].

### Phantom target concentrations

A cohort of 10 mPCa patients that received a [^18^F]DCFPyL PET scan was used to determine activity concentrations for the Probe-IQ phantom (Table 1). These patients are part of a clinical trial (NCT02899312) [4], which was reviewed and approved by the University of British Columbia—BC Cancer Research Ethics Board. Regions-of-interest (ROIs) were placed on the liver, lungs, bladder, ureters, and background compartments (mediastinum, abdomen, and pelvis) (Additional file 1: Fig. S1) using MIM (MIM Software Inc., USA), and activity concentrations were computed for each ROI. Lesion activity concentrations were based on 37 lesions from 33 mPCa patients as delineated by a nuclear medicine physician using a gradient segmentation algorithm (MIM’s PET Edge^+^ tool). The concentration and volume of each lesion were computed.

**Table 1:** Target activity concentrations for compartments in the Probe-IQ phantom

Compartment	Volume (mL)	Activity concentration (kBq/mL)	Relative concentration
Bladder	440 (377 mL)	176.3	98:1
Liver	1025	9.9	5.5:1
Lungs (L)	875	0.67	0.37:1
Lungs (R)	1152	0.67	0.37:1
Pelvis	9000	1.8	1.0:1
Thorax	17,300	1.8	1.0:1
Ureter (L)	10	92.8	51.6:1
Ureter (R)	10	66.5	37:1

### Shell-less ^22^Na lesions

The long-lived positron-emitter, ^22^Na (*t*_1/2_ = 2.6 y), was used as an ^18^F analog for the lesions. ^22^Na and ^18^F have similar positron energy (220.3 keV compared to 252 keV, respectively), which results in similar positron ranges in tissue (0.53 mm vs. 0.6 mm for ^18^F) [25, 26]. The similar positron decay characteristics of both ^22^Na and ^18^F allow for a realistic representation of a [^18^F]DCFPyL PET scan, but provides a significantly longer half-life (2.6 y vs. 109.7 min for ^18^F). The long-lasting ^22^Na lesions have negligible radioactive decay over the duration of multi-hour experiments, providing variable lesion-to-background activity ratios over sequential scans as the lesion remain ~ constant, while the ^18^F background decays. Over 90 spherical lesions (diameters 3–16 mm) were cast using epoxy resin infused with 7.2 kBq/mL, 28.8 kBq/mL, and 57.6 kBq/mL [^22^Na]NaCl, to achieve 4:1, 16:1, and 32:1 lesion-to-background activity ratios. These concentrations were selected to achieve contrast ratios between 5:1 and 17:1, which represent the first and third quartile, respectively, of lesions measured in the patient analysis (Additional file 1: Table S1). In total, 3.7 MBq of activity was required for the ^22^Na casting process. Sphere diameters of 3 mm, 4 mm, 5 mm, 6 mm, 7 mm, 8 mm, 12 mm, 14 mm, and 16 mm were selected, providing a broad range of lesions with realistic contrast and volume (Additional file 1: Fig. S2). In terms of radiation safety, ^22^Na required extensive care and preparation due to its long half-life (*t*_1/2_ = 2.6 y). Prior to ^22^Na lesion casting, multiple practice runs (without activity or using ^18^F as an analog) were performed to ensure that radioactive waste and contamination were minimized. To show that the lesion models were safe to use, the epoxy spheres were submerged in water-filled Falcon tubes prior to the phantom experiment. We then performed wipe tests on the water contained in the Falcon tubes and measured them using a gamma counter. It was verified that ^22^Na contamination did not occur. After the experiment, the ^22^Na waste that was created during preparation was compactly stored in sealable plastic bags in shielded locations, such that they did not occupy too much space. This will be stored for 10 half lives. However, an advantage of the long-lived isotope is that the lesions can be reused for phantom experiments over a long time-period (years), without the need for further lesion casting.

### Image acquisition

Two dynamic PET scans were acquired on a GE Discovery 690 PET/CT scanner (General Electric, USA). The scans were performed using our [^18^F]DCFPyL clinical settings (i.e. the isotope settings correspond to ^18^F).

#### First phantom scan

**Fig. 2:**
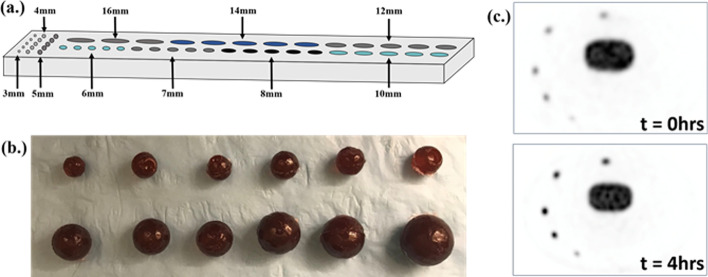
Sodium-22 epoxy spheres. **a** Schematic of aluminium mould used for casting 3–16-mm spheres. **b** Radioactive epoxy spheres (3–16 mm) infused with ^22^Na-NaCl. **c** Transaxial PET image slices of Probe-IQ pelvis with ^22^Na spheres inserted into [^18^F]FDG background, which establishes increased lesion contrast at later times

Eighteen “shell-less” [^22^Na]NaCl epoxy lesions (9 sizes × 2 concentrations) were distributed throughout the Probe-IQ pelvis (Fig. 2). 3–16-mm lesions with two radioactivity concentrations (28.8 kBq/mL and 57.6 kBq/mL) were placed 50 mm from the radial centre of the phantom (Fig. 2c). Lesions of equal concentration were co-planar in two different transaxial slices. The pelvis background and bladder were injected with 65 MBq and 140 MBq [^18^F]fluorodeoxyglucose ([^18^F]FDG), respectively, to achieve target concentrations from the patient analysis (Table 1). A fifteen-frame dynamic acquisition over an 8-h period was performed, adjusting the frame duration to obtain similar counting statistics from [^18^F]FDG in each frame (Table 2). Inter-frame decay correction [27] was applied to account for radioactive decay of the ^18^F. Since the half-life of ^22^Na is much greater than ^18^F the lesion concentration remains relatively constant, while the background decays frame-to-frame. A 2.5-min frame (frame 6 of Table 2) was acquired when the background concentration approximated soft-tissue concentrations observed in PSMA scans (1.8 kBq/mL). This frame duration was selected to agree with our clinical protocol from the patient analysis. The ^22^Na activity concentration ground truth was determined by scanning the lesions after the [^18^F]FDG background fully decayed, 3 days after the dynamic scan, without any repositioning of the phantom.

**Table 2:** Scan protocol using Probe-IQ pelvis (first scan)

Frame number	Frame duration [s]	Background conc. [kBq/mL]	Lesion-to-background ratio
1	60	4.4	13.2
2	72	3.6	16.1
3	87	2.9	19.6
4	105	2.4	24.0
5	127	2.0	29.3
6	153	1.6	36.0
7	185	1.3	44.2
8	223	1.1	54.6
9	270	0.85	67.7
10	326	0.68	84.5
11	394	0.54	106.0
12	476	0.43	134.1
13	575	0.34	171.3
14	695	0.26	221.4
15	839	0.20	290.0

#### Second phantom scan

**Fig. 3:**
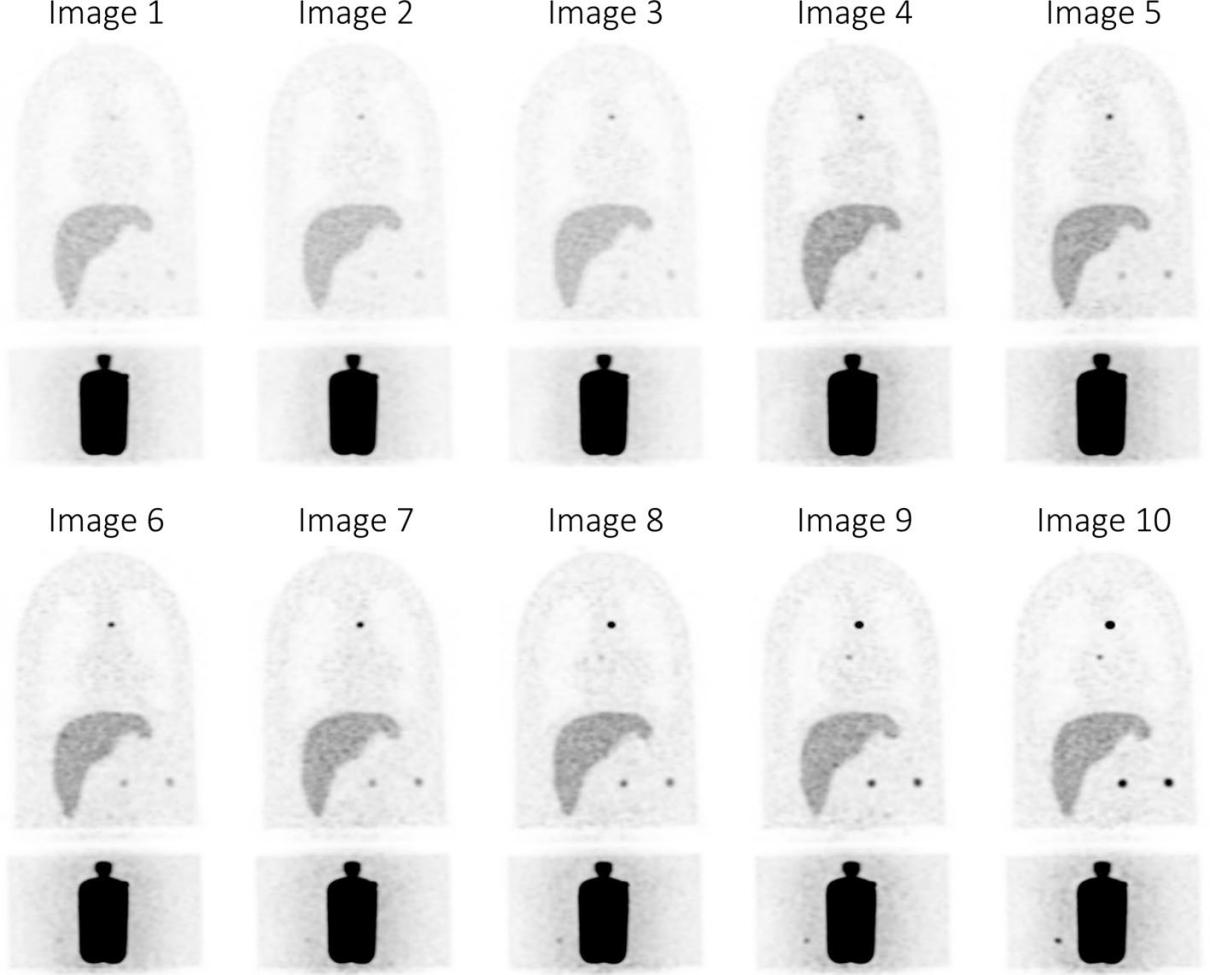
Maximum intensity projection (MIP) images of the anthropomorphic Probe-IQ phantom. The images show increasing lesion contrast as the ^18^F radioactivity in the background decayed (from image 1 to image 10), and the ^22^Na lesion radioactivity remained approximately constant

**Fig. 4:**
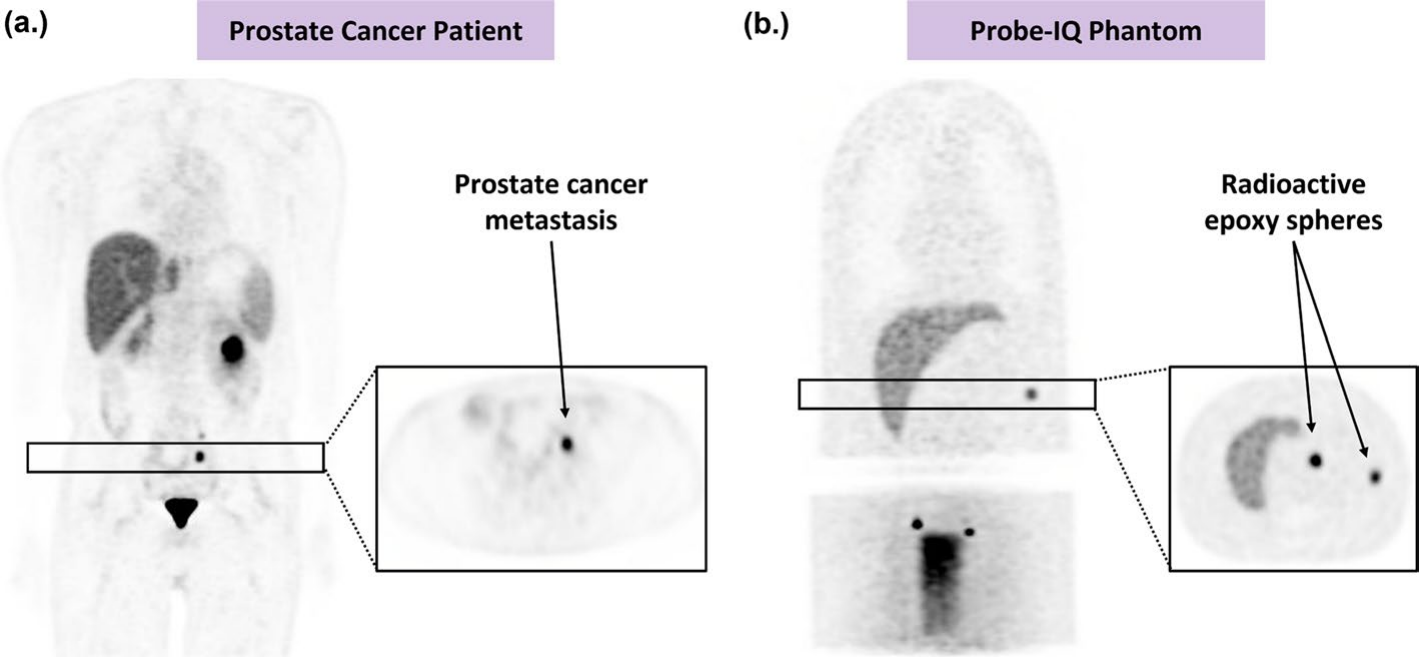
PET images comparing real and simulated lesions in PSMA patient and Probe-IQ phantom, respectively. **a** PSMA PET image of patient with prostate cancer metastasis. **b** PET image of Probe-IQ phantom with embedded radioactive epoxy spheres. Transaxial slices are shown to highlight the realism of the simulated lesions

Twenty-seven lesions (9 sizes × 3 concentrations) were distributed throughout the Probe-IQ phantom (12 in pelvis, five in mediastinum, six in abdomen, four in lungs). 3–16-mm lesion diameters and three concentrations (7.2 kBq/mL, 28.8 kBq/mL, and 57.6 kBq/mL) were selected. Ten whole-body images were obtained over an 8-h period (Figs. 3, 4b). Since the Probe-IQ thorax represents a 92-kg patient [18], longer bed durations (3 min) were selected to ensure sufficient count statistics. Frame and bed durations were decay-corrected to the frame corresponding to PSMA background concentration levels (1.8 kBq/mL). Inter-frame decay correction was applied. ^22^Na-lesion activity concentration ground truth was determined from a 10-min scan with fully decayed background; performed 2 days after the dynamic scan.

### Data analysis

The Probe-IQ pelvis images were reconstructed using ordered subsets expectation maximization (OSEM) (24,32 subsets; 1, 2, 3, 4, 5, 25 iterations) as well as block sequential regularized expectation maximization (BSREM) (32 subsets; 25 iterations; *γ* = 2, *β* = 0, 50, 100, 150, 200, 250, 300, 400, 500, 650, 800) using offline tools provided by General Electric (GE). The BSREM image reconstruction algorithm utilizes a Relative Difference Penalty (RDP), $R\left( x \right)$, which is inserted into the cost function:

$$\hat{x} = \arg \max_{x \ge 0} \mathop \sum \limits_{i = 1}^{{y_{i} }} \log \left[ {P_{x} } \right]_{i} - \left[ {P_{x} } \right]_{i} - \beta R\left( x \right)$$

The RDP is scaled using the *β* value (a smoothing factor), allowing users to select their desired level of noise suppression. General Electric (GE) advertises their BSREM algorithm as Q.Clear [28], and it uses a default number of 25 iterations; 10–13 times higher than regular OSEM. Point spread function (PSF) modelling is included with the Q.Clear algorithm and cannot be disabled in the clinical scanner console. For consistency, PSF modelling was also applied to the OSEM reconstructions. No post-reconstruction filters were applied.

Findings from the first scan (Probe-IQ pelvis) were used to select reconstruction parameters for the second scan (Probe-IQ pelvis and thorax) and patient analysis. Lesion ROIs were drawn with MIM using a 40% of SUV_max_ fixed threshold (40% FT) and a gradient-based (MIM’s PET Edge^+^ tool) segmentation methods. Fixed threshold segmentation selects voxels with concentration greater than a certain threshold (e.g. % of SUV_max_). For the gradient segmentation tool, the user selects a single interior point in the lesion. High activity regions are identified using a contouring algorithm, and tumour boundaries are optimized by computing the second spatial derivative (e.g. inflection points) along concentration line profiles [29, 30]. The gradient-based algorithm performs an interpolation that subdivides the voxel, while the fixed thresholding algorithm utilizes whole voxels.

The ^22^Na ground truth activity concentration ${a}_{h,{\rm true}}$ was determined by dividing the total activity (40-mm spherical ROI) by the known volume on the scan performed without background. Metabolic tumour volume (MTV) and activity recovery coefficients (max, mean, peak, and apex) were calculated for each sphere size. Recovery coefficients were calculated using the following formula:

$${\text{RC}} = \frac{{a_{{h,{\text{meas}}}} }}{{a_{{h,{\text{true}}}} }} \times 100$$

where $a_{{h,{\text{meas}}}}$ may refer to the lesion max, mean, peak, or apex (defined below) activity concentration. We also defined total tumour uptake (TTU), given by the product of the mean activity concentration and MTV:

$${\text{TTU}} = a_{{h,{\text{mean}}}} \times {\text{MTV}}$$

Since the physical volume of most lesions was less than 1 mL, an alternate method to SUV_peak_, which we denote by SUV_apex_, was also developed. We defined SUV_apex_ as the mean concentration of a spherical ROI centred at the SUV_max_ voxel. The ROI is interpolated to enclose a volume equal to 0.26 mL (approximately 6 voxels). The VOI volume was selected such that it would be small enough to quantify focal lesions that are characteristically observed in PSMA PET, but large enough such that precision loss due to image noise was minimized by averaging over six voxels.

Mean absolute error (MAE), as a measure of accuracy, was defined as:

$${\text{MAE}} = \frac{{\mathop \sum \nolimits_{i}^{n} \left| {a_{i} - a_{{h,{\text{true}}}} } \right|}}{n}$$

in which a series of $n$ measurements of activity concentration, $a_{i} \left( {a_{{h,{\text{meas}}}} } \right)$, is computed for a given segmentation method.

Sample standard deviation was used as a measure of precision:

$$s = \sqrt {\frac{{\mathop \sum \nolimits_{i}^{n} \left( {a_{i} - \overline{a}} \right)^{2} }}{n - 1}}$$

in which a series of $n$ measurements of activity concentration, $a_{i} \left( {a_{{h,{\text{meas}}}} } \right)$, and mean activity, $\overline{a}$, is computed between data points of all reconstruction parameters, for a given segmentation method.

To find statistical differences between each metric (e.g. MTV values using threshold vs. gradient segmentation), paired t tests were performed:

$$t = \frac{{\left| {m_{1} - m_{2} } \right|}}{s/\sqrt n }$$

in which $m_{1}$ and $m_{2}$ are the mean differences, $s$ is the sample standard deviation, and $n$ is the sample size.

Contrast was defined in two ways; one that uses the maximum activity concentration and another that uses the mean activity concentration as shown below:

$$C_{\max } = \frac{{a_{h,\max } }}{{a_{{{\text{bkg}}}} }} \times 100$$

$$C_{{{\text{mean}}}} = \frac{{a_{{h,{\text{mean}}}} }}{{a_{{{\text{bkg}}}} }} \times 100$$

where $a_{{h,{\text{max}}}}$ and $a_{{h,{\text{mean}}}}$ are the average and maximum activity concentration in an ROI. $a_{{{\text{bkg}}}}$ is the background concentration.

The contrast-to-noise (CNR) and signal-to-noise ratios (SNR) of lesions were also evaluated:

$$\begin{aligned} & {\text{CNR}}_{\max } = \frac{{a_{h,\max } - a_{{{\text{bkg}}}} }}{{\sigma_{{{\text{bkg}}}} }}\quad {\text{SNR}}_{\max } = \frac{{a_{h,\max } }}{{\sigma_{{{\text{bkg}}}} }} \\ & {\text{CNR}}_{{{\text{mean}}}} = \frac{{a_{{h,{\text{mean}}}} - a_{{{\text{bkg}}}} }}{{\sigma_{{{\text{bkg}}}} }}\quad {\text{SNR}}_{{{\text{mean}}}} = \frac{{a_{{h,{\text{mean}}}} }}{{\sigma_{{{\text{bkg}}}} }} \\ \end{aligned}$$

where $a_{h,\max }$ and $a_{{h,{\text{mean}}}}$ are the average and maximum activity concentrations in an ROI, respectively. $a_{{{\text{bkg}}}}$ is the background concentration in a 0.5 mL ROI, and $\sigma_{{{\text{bkg}}}}$ is the standard deviation of five $a_{{{\text{bkg}}}}$ measurements.

## Results

### Lesion segmentations

**Fig. 5:**
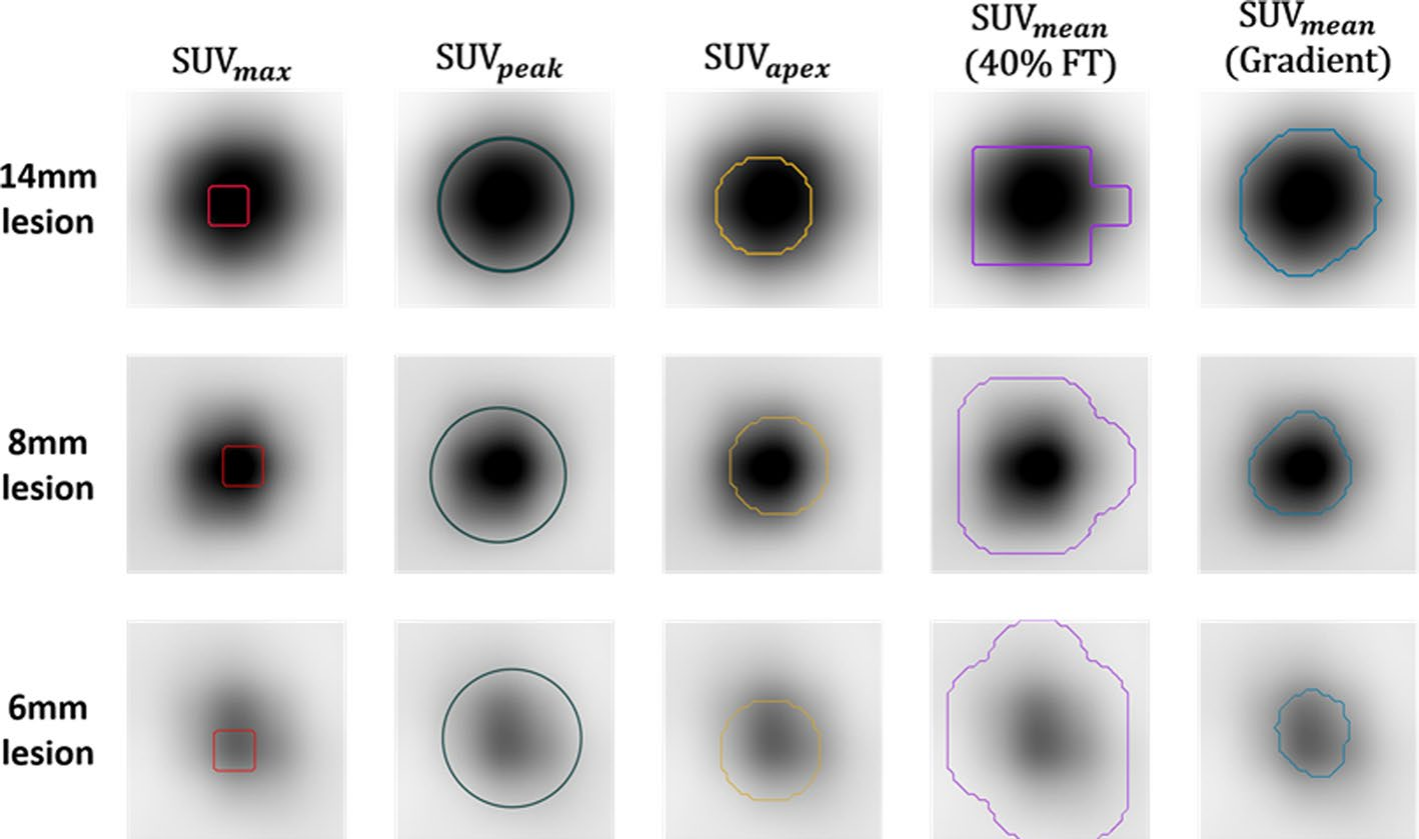
SUV metrics applied to simulated lesions in the Probe-IQ phantom. Max, peak, apex, 40% FT, and gradient methods applied to PET images of 14-mm, 8-mm, and 6-mm ^22^Na epoxy spheres reconstructed with OSEM (32 subsets, two iterations)

Figure 5 shows lesions segmented in the Probe-IQ pelvis. It can be observed that the 1-mL ROI defined by SUV_peak_ is too large for the 6-mm and 8-mm lesions. Meanwhile, the SUV_max_ metric only measures a single voxel in the lesion. SUV_apex_ defines a volume that is intermediate to the previous two methods (0.26 mL or approximately six voxels), which corresponds to an appropriate ROI size for the 8-mm and 14-mm lesions. Segmentation using 40% FT overestimated the 6-mm and 8-mm lesion boundaries, while the gradient method smoothly followed the boundary of each lesion.

### Recovery curves

**Fig. 6:**
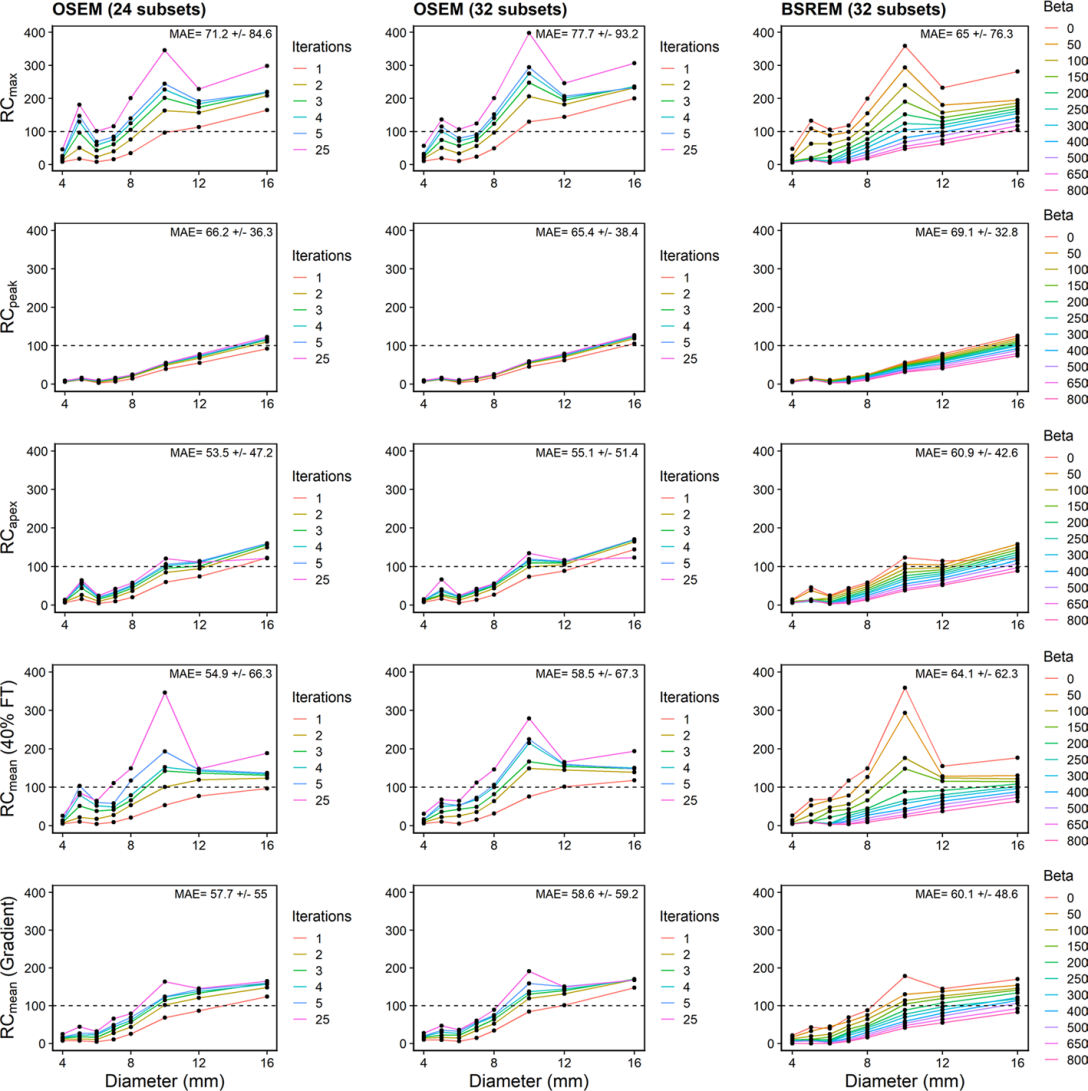
Recovery curves. (Top to bottom) Recovery concentration coefficients measured in Probe-IQ pelvis using Max, Peak, Apex, and Mean (40% FT and gradient). (Left to right) Reconstruction algorithms using OSEM + PSF (24 and 32 subsets, respectively) and BSREM. Mean absolute error (MAE) ± Standard Deviation indicated on each plot

Recovery coefficients (RCs) were plotted to evaluate the accuracy of each reconstruction algorithm and segmentation method (Fig. 6). SUV_max_ (shown as RC_max_ in plots) resulted in overestimated RCs that peaked for the 10-mm sphere. This effect was amplified at higher number of iterations for OSEM (294.8% for 32 subsets, five iterations) and lower *β* values for BSREM (293.9% for *β* = 50). Recovery overestimation was prevented by using only one iteration (OSEM) or *β* $\ge$ 400 (BSREM). RCs were underestimated for lesions smaller than 10 mm, and *β* < 200 was required for BSREM to minimize recovery loss through signal smoothing (25.4% and 77.9% for 5 mm and 7 mm, respectively). For OSEM, at least three iterations were required to improve the concentration recovery (114.9% and 91.4% for 5 mm and 7 mm, respectively, 32 subsets). The SUV_peak_ recovery curve followed a monotonic, increasing relationship with respect to lesion diameter. For OSEM (32 subsets, two iterations), the 8-mm, 10-mm, 12-mm, and 16-mm lesions had 23.2%, 54.6%, 71.5%, and 118.7% recovery. Meanwhile, for BSREM (*β* = 300), the same spheres had recovery coefficients of 18.7%, 43.2%, 58.7%, and 99.3%. The SUV_apex_ recovery curves sharply increased from 6 to 10 mm, and plateaued in the 10–12-mm range. The 10-mm and 12-mm lesion results were most accurate using BSREM *β* = 100 (89.9% and 97.8% respectively), and OSEM with two iterations (99.3% and 105.3%, using 32 subsets).

SUV_mean_ using 40% FT was most accurate using BSREM *β* = 200 (88.4%, 91.9%, and 108.7% for 10 mm, 12 mm, and 16 mm, respectively). BSREM with lower *β* values minimized signal loss for smaller lesions (12.3%, 24.5%, and 64.1% for 4 mm, 6 mm, and 8 mm, respectively, using *β* = 100). For OSEM with 32 subsets, the most accurate recovery for lesions greater than 10 mm was achieved after only one iteration (75.7%, 101.5%, 118.0% for 10 mm, 12 mm, and 16 mm, respectively). OSEM with 24 subsets resulted in comparatively lower recovery values at one iteration (53.1%, 77.0%, 97.2% for 10 mm, 12 mm, and 16 mm, respectively). Higher numbers of iterations (4 or 5) were needed to minimize signal loss for smaller lesions (4–8 mm). Gradient segmentation was most accurate using BSREM with *β* = 300 for larger lesions (69.5%, 89.2%, and 121.9% for 10 mm, 12 mm, and 16 mm respectively). *β* = 50 was required to increase recovery for smaller lesions (17.1%, 45.0%, 74.7% for 4 mm, 6 mm, and 8 mm, respectively). OSEM was most accurate after 1 iteration for 12–16-mm lesions (84.2%, 100.9%, 147.6% for 10 mm, 12 mm, and 16 mm, respectively). Smaller lesions were underestimated with OSEM, so higher iterations were needed to maximize signal recovery. Overall, SUV_peak_ had lower mean absolute error than SUV_max_. SUV_apex_, a hybrid between SUV_max_ and SUV_peak_, significantly reduced MAE for both reconstruction algorithms (OSEM and BSREM were 55.1 $\pm$ 51.4% and 60.9 $\pm$ 42.6%, respectively). Gradient and 40% FT segmentation had similar MAE as SUV_apex_.

### Robustness of recovery curves

**Fig. 7:**
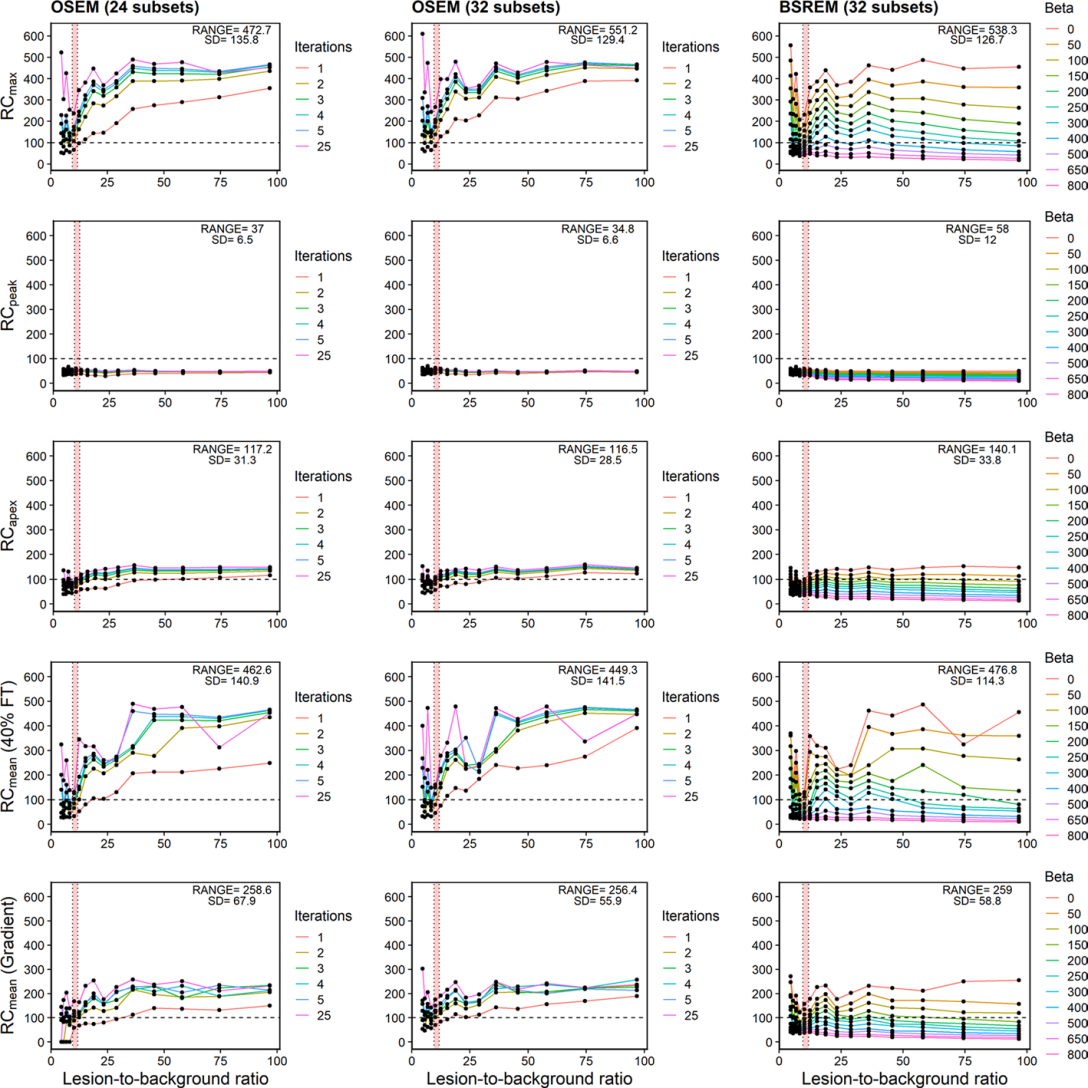
Robustness of recovery curves. Recovery concentration coefficient versus lesion-to-background ratio for 10-mm lesion measured in Probe-IQ pelvis. (Top to bottom) Max, Peak, Apex, and Mean (40% FT and gradient). (Left to right) Reconstruction algorithms using OSEM + PSF (24 and 32 subsets, respectively) and BSREM. Range and standard deviation of recovery coefficients annotated on plots. [^18^F]DCFPyL background activity levels are represented by red shaded region

RCs were plotted versus lesion-to-background ratios from different frames of the dynamic scan (Fig. 7, Additional file 1: Fig. S3 to S9). The pink shaded regions in the figure indicates when the phantom background activity entered the range of typical PSMA concentrations. The 10-mm lesion was observed to be most unstable versus lesion-to-background ratio, so it was deemed to be most useful for observing differences between each segmentation method (Fig. 7). Standard deviation of RC values was computed between all points on each plot. This allowed us to evaluate the consistency of each metric for different reconstruction parameters and lesion-to-background ratios. For both OSEM and BSREM (32 subsets), standard deviation was the highest using SUV_max_ (551.2%, and 538.3%) and 40% FT (449.3% and 476.8%). By comparison, standard deviation using gradient segmentation was lower and RCs appeared to be less dependent on the reconstruction parameter (e.g. *β* value or number of iterations). SUV_peak_ was the most reproducible metric and had standard deviations of only 34.8% and 58.0% for the OSEM and BSREM algorithms, respectively, while SUV_apex_ was the second-most consistent metric (116.5% and 140.1%).

### Tumour volume and uptake

**Fig. 8:**
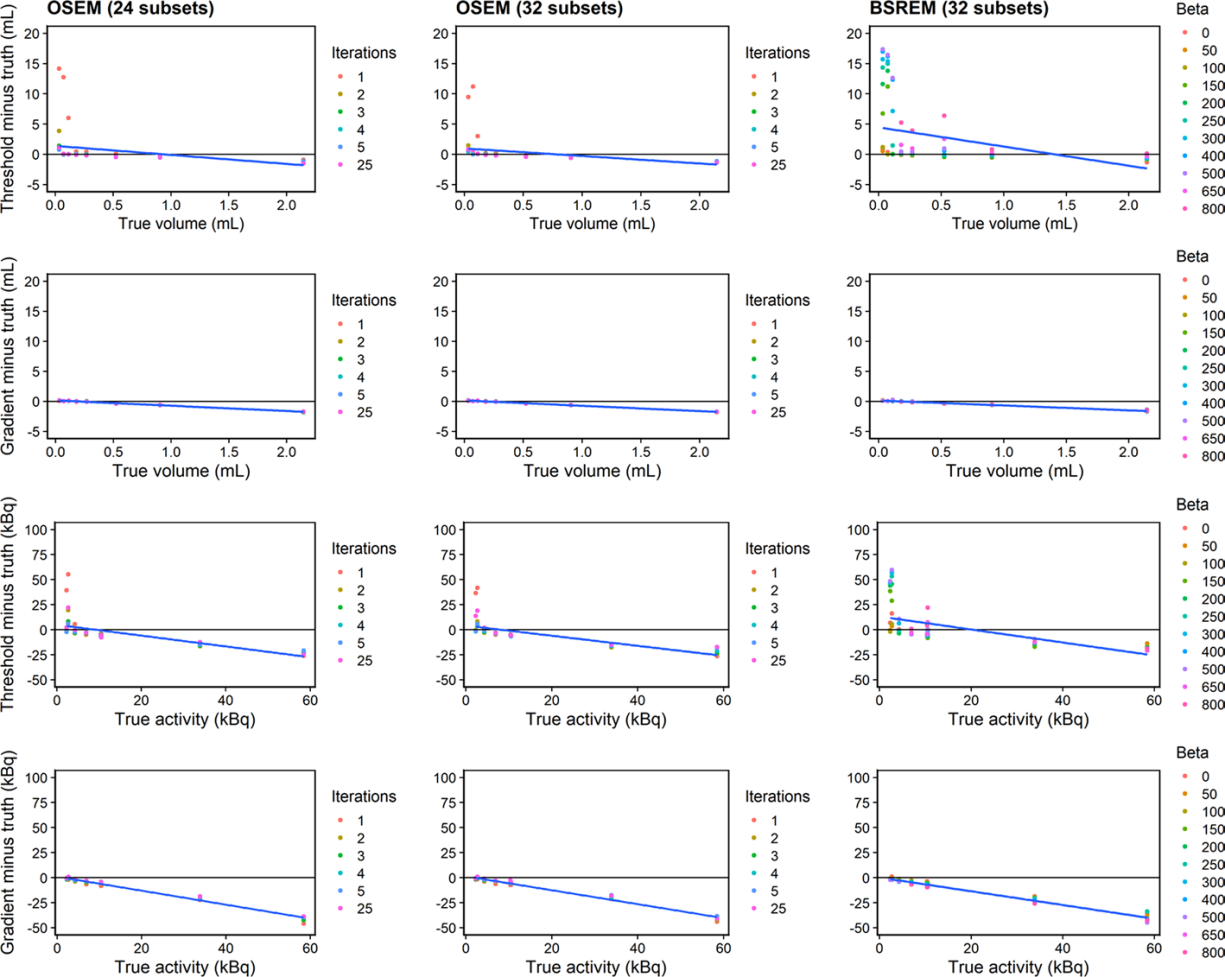
Tumour volume and uptake accuracy using 40% fixed threshold and gradient segmentation. Difference between measured value and ground truth, plotted vs. ground truth, for metabolic tumour volume (MTV) and total tumour uptake (TTU) metrics. (Top to bottom) 40% FT and gradient segmentation. (Left to right) Reconstruction algorithms using OSEM + PSF (24 and 32 subsets respectively) and BSREM. Blue line indicates overall fit

Metabolic tumour volume (MTV) and total tumour uptake (TTU) values were compared with the ground truth using 40% FT and gradient segmentation (Fig. 8). The gradient method appeared to be more accurate for delineating lesion boundaries (Fig. 5), while the 40% FT significantly overestimated MTV for 3–10-mm lesions. MTV and TTU bias for the 40% FT was the largest when using one iteration for OSEM. Linear fits were applied to each plot, and the coefficient of determination was computed (Additional file 1: Table S2). A nonlinear trend was observed while plotting MTV bias vs. ground truth for the 40% FT (*R*^2^ < 0.1), which resulted from MTV overestimation for small sphere sizes. Strong linear correlation while plotting bias vs. ground truth was observed for the gradient segmentation (*R*^2^ $\ge$ 0.987 and *R*^2^ $\ge$ 0.979 for MTV and TTU, respectively), which indicates a consistent trend for segmenting spheres with different reconstruction parameters.

### CNR and SNR measures

**Fig. 9:**
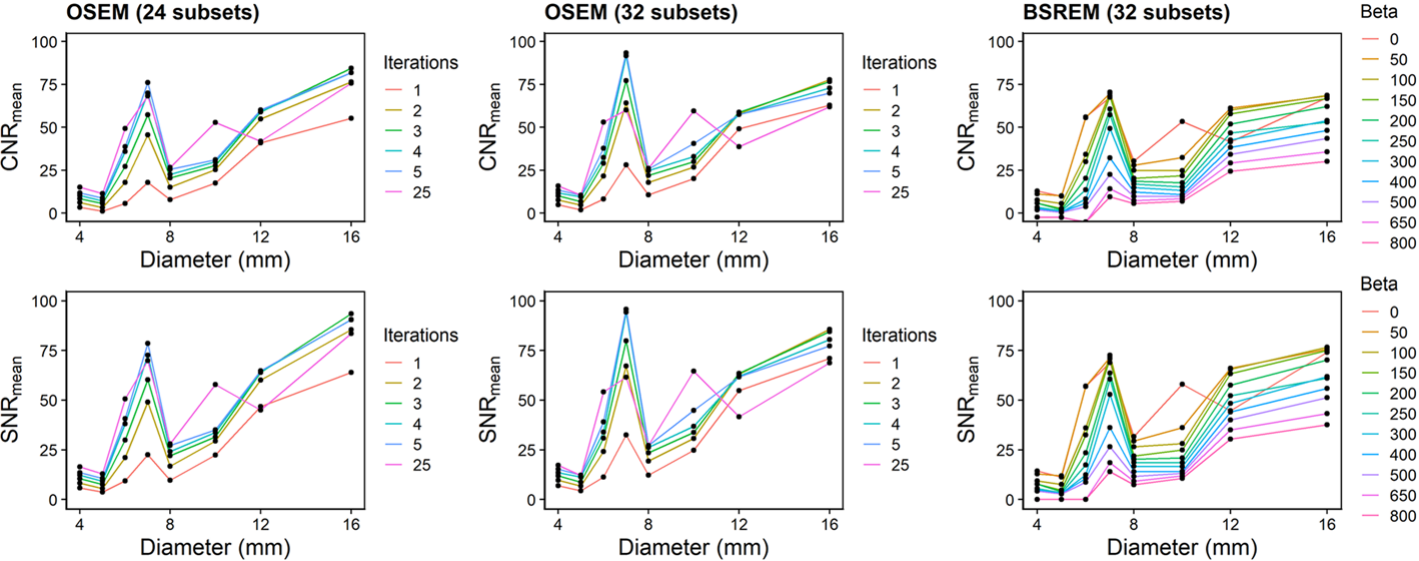
Contrast-to-noise ratio (CNR) and signal-to-noise ratio (SNR) metrics. (Top to bottom) CNR and SNR plotted vs. lesion diameter, using RC_mean_ with gradient segmentation. (Left to right) Reconstruction algorithms using OSEM + PSF (24 and 32 subsets, respectively) and BSREM

Contrast-to-noise ratio (CNR) and signal-to-noise ratio (SNR) were computed using SUV_mean_ with gradient segmentation (Fig. 9). CNR_mean_ values were the highest for 10–16-mm lesions using OSEM with 2–3 iterations (CNR_mean_ = 58.0 for 12 mm with two iterations, 32 subsets). Additional iterations were needed to converge the images and increase CNR_mean_ for < 10-mm lesions. CNR_mean_ values for BSREM were the highest using *β* values between 50 and 300 (CNR_mean_ = 60.1 for 12 mm with *β* = 100). Lower *β* values (e.g. 0 to 100) increased CNR_mean_ for < 10-mm lesions. SNR_mean_ followed similar trends as CNR_mean_, peaking after 2–3 iterations (OSEM) or for *β* values between 50 and 300 (BSREM). Trends for CNR_max_ and SNR_max_ can be found in Additional file 1: Fig. S10.

### Application of selected parameters to Probe-IQ phantom

**Fig. 10:**
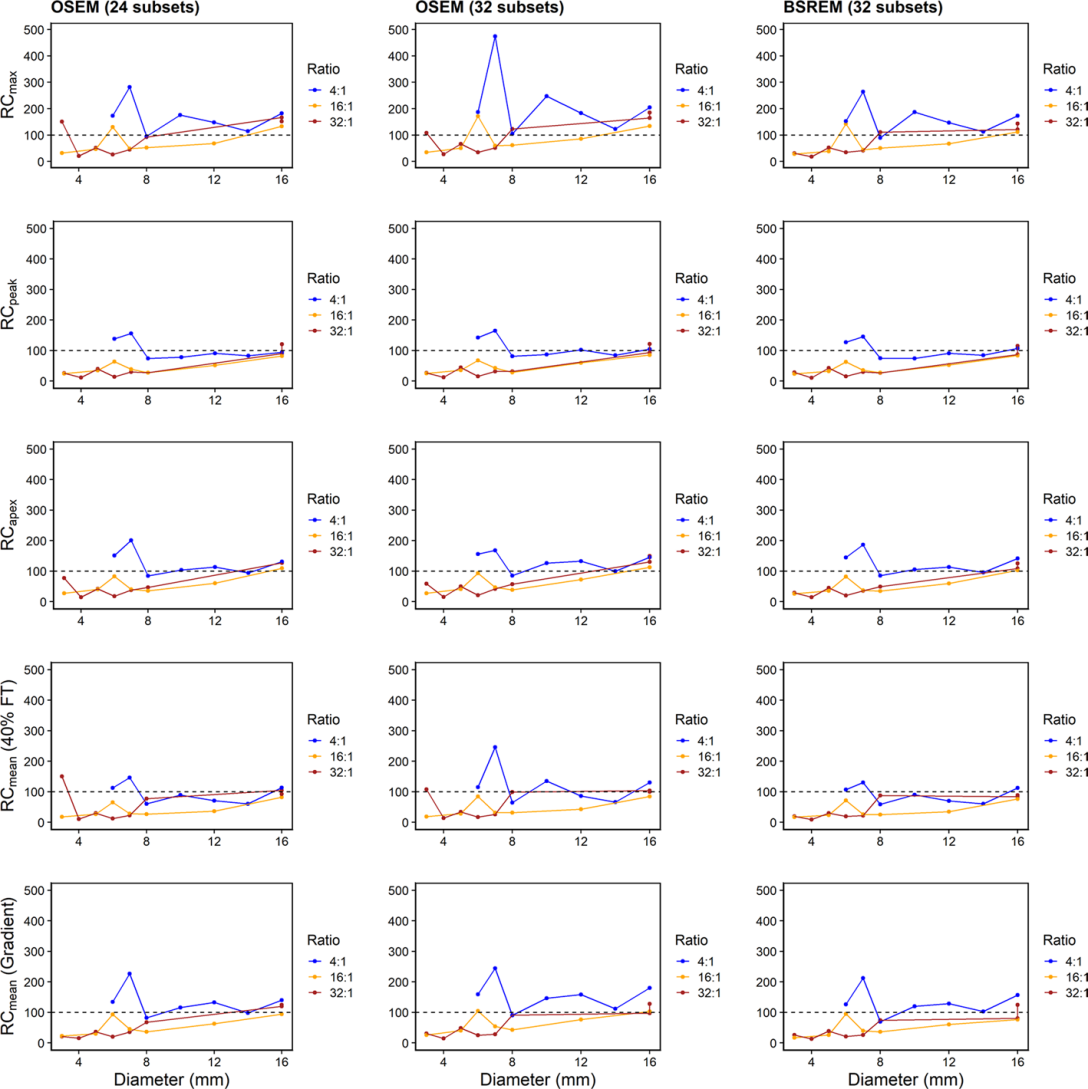
Recovery curves for epoxy spheres with different ^22^Na activity concentrations. (Top to bottom) Probe-IQ recovery concentration coefficients vs. lesion diameter using Max, Peak, Apex, and Mean (40% FT and gradient). (Left to right)—reconstruction algorithms using OSEM + PSF (24 and 32 subsets, respectively) and BSREM. Each colour represents a different lesion-to-background activity ratio

Reconstruction parameters for OSEM (24,32 subsets, two iterations) and BSREM (32 subsets, 25 iterations, *γ* = 2, *β* = 300) were selected to ensure reasonable image quality and quantitation accuracy, based on observations from the first scan. RC values were plotted for each metric (Fig. 10). Spheres infused with higher concentrations of ^22^Na (28.8 kBq/mL and 57.6 kBq/mL) had much lower RCs for 6–12-mm lesions compared to spheres infused with ^22^Na at a concentration of 7.2 kBq/mL. For instance, RC for the 6-mm lesion using SUV_max_ and SUV_mean_ (gradient), respectively, were 247.3% and 160.0% for the low concentration spheres, but only 34.5% and 25.1%, for the spheres with a ^22^Na concentration of 57.6 kBq/mL (OSEM with 32 subsets). In terms of MTV and TTU (Additional file 1: Fig. S11), the 40% FT consistently overestimated volume for < 1 mL lesions. The gradient segmentation underestimated MTV and TTU for larger lesions (e.g. > 1 mL), but was quite accurate as the true volume approached zero, as the y-intercept fit for MTV and TTU was 0.080 mL and 1.48 kBq, respectively, for OSEM with 32 subsets.

### Patient analysis

**Fig. 11:**
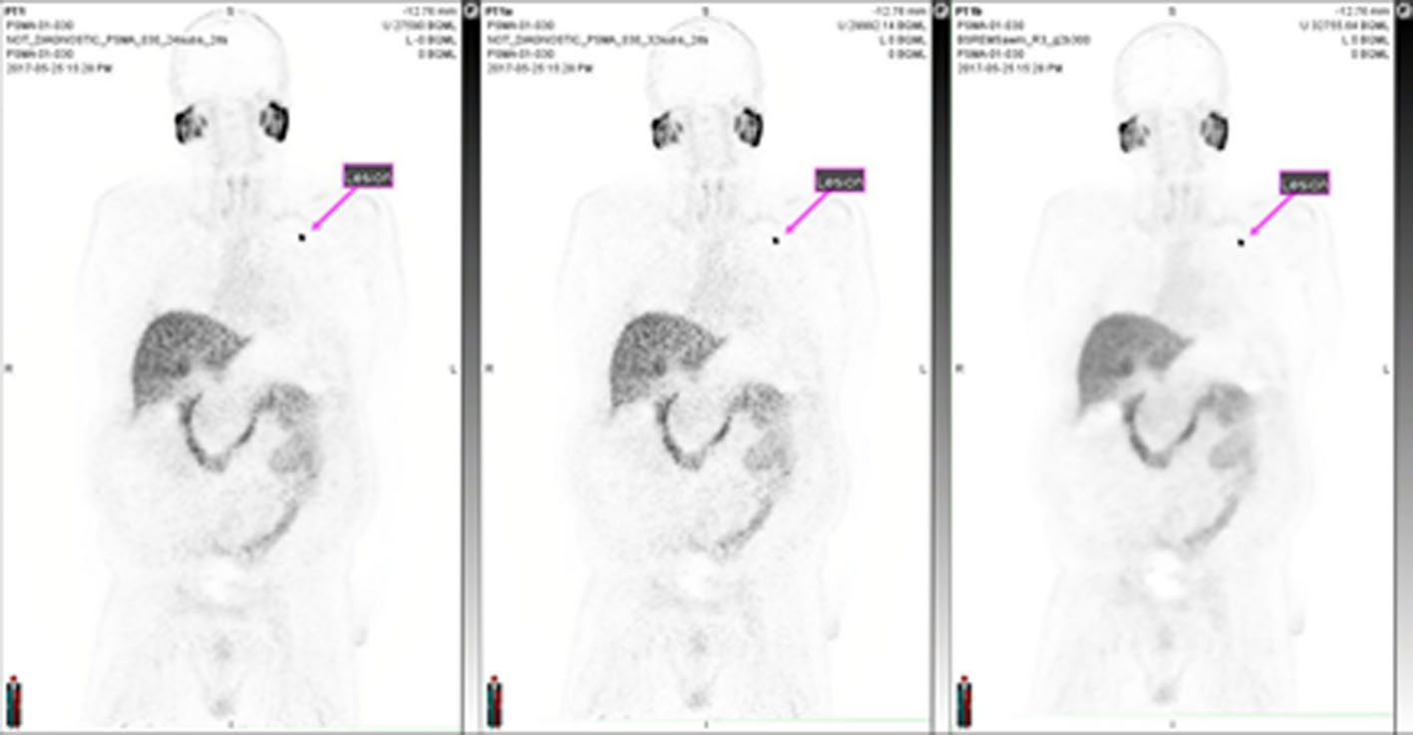
Reconstruction algorithms applied to patient imaged with PSMA PET. (Left to right) [^18^F]DCFPyL PET images reconstructed with OSEM (24 subsets, two iterations), OSEM (32 subsets, two iterations), and BSREM (32 subsets, 25 iterations, *β* = 300)

**Fig. 12:**
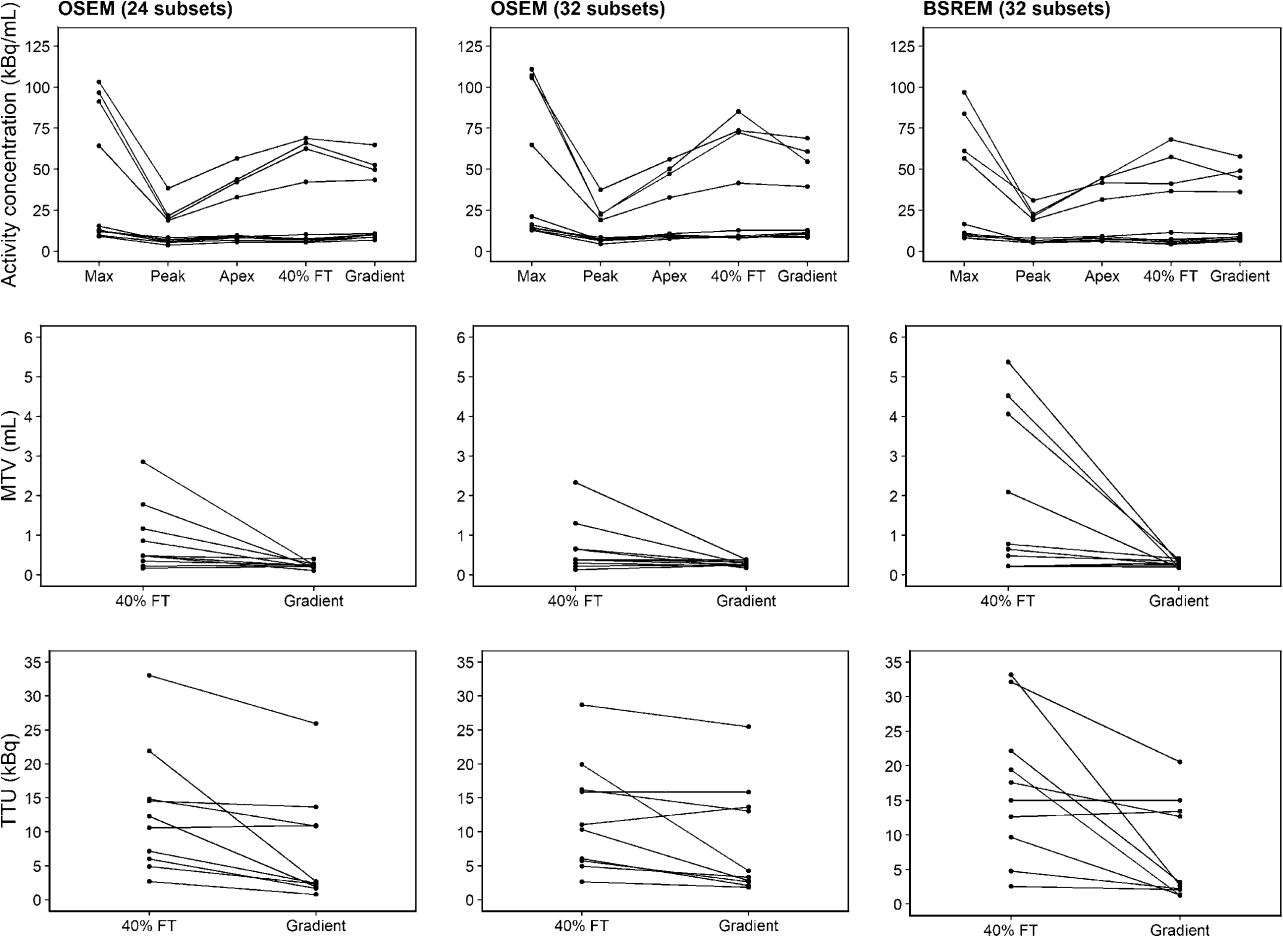
Lesion activity concentration, volume, and uptake determined from patient analysis. Activity concentration plotted using max, peak, apex, mean (40% FT and gradient) metrics (top), metabolic tumour volume; MTV using 40% FT and gradient segmentation (middle row), and total tumour uptake; TTU using 40% FT and gradient segmentation (bottom) for ten lesions from [^18^F]DCFPyL patient images

Ten [^18^F]DCFPyL PET images from the patient analysis were reconstructed and segmented using the same methods described in the phantom study (Fig. 11). Activity concentration and MTV for each metric is shown in Fig. 12, and statistics can be found in Additional file 1: Table S3. Activity concentrations using SUV_max_ had the highest mean value and inter-quartile range (42.7 kBq/mL and 12.3–84.4 kBq/mL, respectively). The lowest mean value and inter-quartile range was observed for SUV_peak_ (13.5 kBq/mL and 5.9–19.6 kBq/mL, respectively). The interquartile range and average concentration for SUV_apex_ were intermediate to those observed for SUV_max_ and SUV_peak_. Compared to gradient segmentation, increased variability in activity concentration and MTV were observed using 40% FT. As shown in Table 3, differences in SUV_mean_ between the gradient and 40% FT segmentation were not statistically significant (0.227 < *P* < 0.8043), as computed using a paired *t* test. However, differences between 40% FT and gradient segmentation were more distinct for the MTV and TTU metrics (0.036 < *P* < 0.083 and 0.014 < *P* < 0.045, respectively).

**Table 3:** Mean difference between methods for determining lesion activity concentration (kBq/mL), MTV (mL), and TTU (kBq)

Metric	Method	OSEM (24 subsets)	OSEM (32 subsets)	BSREM (32 subsets)
Concentration	Max versus Peak	29.19, ***p* = 0.0156**	33.76, ***p* = 0.0129**	23.22, ***p* = 0.0232**
Concentration	Max versus Apex	20.29, ***p* = 0.0167**	23.72, ***p* = 0.0132**	15.38, ***p* = 0.0256**
Concentration	Peak versus Apex	-8.90, ***p* = 0.0135**	-10.04, ***p* = 0.0125**	-7.84, ***p* = 0.0202**
Concentration	Threshold versus Gradient	1.71, *p* = 0.418	4.19, *p* = 0.227	0.55, *p* = 0.804
MTV	Threshold versus Gradient	0.646, ***p* = 0.0400**	0.394, *p* = 0.0829	1.576, ***p* = 0.0362**
TTU	Threshold versus Gradient	5.47 ***p* = 0.0144**	3.62 ***p* = 0.0450**	9.46 ***p* = 0.0176**

*P*-value from a paired *t* test is shown (bolded values indicate significance below *α* = 0.05)

## Discussion

Harmonization of clinical trials can enable development of robust multi-centre studies and lead to improved predictive modelling for prostate cancer [31–34]. Given the increasing number of clinical trials using PSMA PET [4, 5, 35–38], there is significant motivation to validate quantitative metrics for different lesion sizes and reconstruction parameters. In this study, we aimed to evaluate PSMA PET quantitation by performing a phantom study using radioactive epoxy spheres to simulate lesions with known ground truth. To represent prostate cancer metastasis, we cast small spheres (3–16-mm diameter) with high radioactivity (57.6 kBq/mL) to represent the focal, high-contrast lesions that are frequently observed in PSMA PET/CT scans (Fig. 4). The study was done using Probe-IQ, an anthropomorphic phantom that has been previously used in lesion detectability [18, 19, 24] and quantitation studies [16, 17]. Phantom inserts were used to simulate the organs that typically show uptake in PSMA PET, including a liver and bladder, as well as ureters specifically designed for this study. Polyurethane filter foam was used to position lesions within the phantom and create small pockets of air to establish heterogeneous radioactivity distributions, which is a more realistic representation of a patient image compared to a uniform phantom [10, 20].

We do recognize some limitations within this study. Since [^18^F]DCFPyL is the PSMA-based agent used at our institution (BC Cancer), we used ^22^Na to model lesions imaged with ^18^F-PSMA tracers. However, these results do not generalize to all PSMA-based tracers, such as ^68^Ga-PSMA agents that are frequently used in the clinical setting [3]. Similar phantom experiments need to be developed to specifically evaluate ^68^Ga-PSMA imaging. Within this study, we were limited by the number of epoxy spheres and radioactivity concentrations, as well as their locations within the phantom. ^22^Na has a high energy gamma line (1274 keV) representing 9.5% of decay events, which can generate scattered photons in the energy window of the PET scanner and that can potentially increase to number of random coincidence detection. However, as the ^22^Na lesions represented a small relative amount of total activity in the phantom (for the first phantom scan this was < 1 MBq of ^22^Na vs. 205 MBq of ^18^F in the lesions and background, respectively), we expect this to have a minor effect. Since the ground truth was determined using a long duration scan (10 min) without background, this allowed us to minimize quantitative differences resulting from the high energy gamma line in the ^22^Na lesions.

Lesion casting took four 3-h sessions, and the Probe-IQ phantom required 4 h of preparation time. This is much more radioactivity handling time than a typical NEMA phantom. As a result, personnel receive a higher radiation dose due to (1) the higher ^18^F activity used to fill the phantom to account for the decay in a longer preparation time, and (2) due to being exposed for a longer time. Radiation protection measures were implemented, such as placing a lead wall shield in between the phantom and the researcher while preparing the phantom (e.g. tightening screws). We also tried to keep distance between the phantom and research team as large as possible. The measured dose to the hands and torso from conducting the experiment were 200 μSv to the hands (measured with thermoluminescent ring dosimeters) and 25 μSv to the torso, as measured with an electronic personal dosimeter (EPD). For reference, our usual torso dose for NEMA phantom preparation is approximately 9 μSv. The large size of Probe-IQ restricts its use to in-house experiments, and limits its ability to ship the phantom within large-scale, multi-centre imaging studies. Another disadvantage of the Probe-IQ phantom is that it does not easily account for anatomical variation in the patient population. Virtual clinical trials (VCTs) [39–43], in which physical phantoms are replaced with digital phantoms, provide the opportunity to efficiently scale demographic features such as patient height, weight, or organ uptake. It is important that subsequent studies validate our findings for use in the broader patient population.

It is well-known that definitions of SUV (e.g. max, mean, peak) can impact quantitation and clinical interpretation of treatment response in PET [44]. A variety of segmentation metrics have been proposed and evaluated for prostate cancer [44, 45]; however, many remain to be properly validated within this emerging context of PSMA. In our study, the accuracy and precision of recovery coefficients (Figs. 5 and 6, respectively) was compared for different SUV and MTV metrics. The poor reproducibility of SUV_max_ for different lesion-to-background ratios can be attributed to Poisson noise on single-voxel metrics (Fig. 6) [46, 47]. The overestimated recovery curve that peaked at 10 mm (Fig. 5), most certainly caused by Gibbs ringing associated with the PSF modelling, is in agreement with findings by Kaalep et al. [47]. This effect is problematic, since a 10-mm lesion would potentially exhibit a higher SUV_max_ value than a larger lesion of the same concentration. Analogous to observations by Kaalep et al., in which a carefully selected post-reconstruction filter can minimize recovery overestimation, our results suggest that through careful selection of reconstruction parameters (e.g. higher noise-penalization factors for BSREM or fewer reconstruction iterations for OSEM), overestimation of RCs in small lesions can be minimized.

Compared to SUV_max_, we found that RCs measured using SUV_peak_ were more consistent for different lesion-to-background ratios. Although SUV_peak_ underestimated concentration for smaller lesions (e.g. $\le$ 12-mm diameter), its consistency suggests that it may still be considered useful for clinical practice. Reduced variability of lesion concentration due to image reconstruction parameters may result in improved comparison between different imaging centres. Our newly defined metric, SUV_apex_, was developed with the intention of increasing RCs for smaller lesions, while minimizing variability resulting from different reconstruction parameters. SUV_apex_ improved accuracy for 10–12-mm lesions, assuming that a reasonable *β* value was selected for BSREM (e.g. *β* = 100–400). RC overestimation for the 10-mm lesion, notably present with SUV_max_, was not observed while using SUV_apex_. In terms of robustness, SUV_apex_ had low variability for different lesion-to-background ratios and reconstruction parameters. Therefore, SUV_apex_ appears to be a potential “happy medium” solution between the SUV_max_ and SUV_peak_ metrics. Further research is needed to evaluate additional variations of SUV_peak_ for quantification of PSMA lesions. These variations may include different contour sizes, shapes (e.g. spherical versus circular), and localization (e.g. centred on SUV_max_ versus finding the highest average uptake region) [44].

Lesion concentration, volume, and uptake were evaluated using two segmentation methods (40% FT and gradient) to calculate SUV_mean_. For our imaging conditions, we found that RCs using the 40% FT were most accurate using *β* = 200–400 for BSREM or one iteration for OSEM. However, as observed visually, images reconstructed with only one iteration of OSEM were not properly converged, which suggests that this selection of reconstruction parameter should not be used. Similar to SUV_max_, RC overestimation was also observed for certain reconstruction parameters (e.g. greater number of iterations or lower *β* values). Overall, the 40% FT does not appear to be very robust, as it was severely influenced by the reconstruction parameters and lesion-to-background ratios. By comparison, the gradient segmentation method was effective at minimizing the recovery peak at 10 mm and was more consistent for different lesion-to-background ratios. Given a reasonable selection of reconstruction parameters (e.g. *β* = 200–400 for BSREM and 2–5 iterations for OSEM), the gradient and 40% FT segmentation provided reasonably accurate SUV values. In terms of lesion volume, both segmentation methods underestimated MTV for 10–16-mm diameter lesions. However, the 40% FT was less accurate for smaller lesions. This occurred as the lesion size approached the scanner resolution limit and the partial volume effect (PVE) reduced the observed contrast of the tumours (Fig. 8). This was particularly evident for reconstruction parameters with incomplete image convergence (e.g. OSEM with one iteration) or over-smoothed images (e.g. BSREM with *β* $\ge$ 200) which resulted in the 40% FT selecting voxels in the background. The gradient method was more robust to reconstruction parameter selection, since the method was based on line profile concentration gradients [29, 30], rather than selecting voxels with concentrations that contain 40% or greater of the maximum uptake.

Based on our findings, SUV_max_ does not appear to be an appropriate metric for quantification of PSMA PET images. Rather, we believe that PSMA quantitative parameters should be based on SUV_peak_ or related metrics (e.g. SUV_apex_). SUV_peak_ was the most robust versus lesion-to-background ratio but RCs were underestimated for focal lesions because the 1-mL ROI was too large. SUV_apex_ appears to somewhat resolve this issue by utilizing a smaller ROI. To determine lesion volume, we found that the gradient segmentation was much more accurate than the 40% FT.

By infusing the epoxy with different concentrations of ^22^Na, we were able to evaluate how quantitation may vary for different lesion concentrations (Fig. 10). The high-concentration spheres (resulting in 16:1 and 32:1 lesion-to-background ratios) represented lesions imaged with PSMA PET (Additional file 1: Table S1), while the lowest concentration spheres (4:1) are representative of lesions imaged with conventional PET tracers, such as [^18^F]FDG. RCs for the [^18^F]FDG and PSMA spheres had greater differences for smaller spheres (6–12 mm). The PSMA spheres underestimated lesion concentration more significantly than the [^18^F]FDG spheres, which was likely caused by the spill-out effect [48–50]. These findings highlight the importance of validating PSMA tracers with phantom studies, as our results differed dramatically from conventional imaging paradigms.

As shown in this phantom study, we found that comparable quantitative accuracy can be achieved for PSMA using either the OSEM or BSREM reconstruction algorithm. However, the primary advantage of BSREM can be attributed to its improved lesion detection and image quality [22, 23]. The “smoothing factor” in the cost function ensures that noise is suppressed, which allows for significantly more reconstruction iterations and improved image convergence. Larger *β* parameters reduce image noise, but also penalizes large concentration gradients; this may reduce the observed contrast of lesions. Care must be taken to configure reconstruction parameters for both the purposes of image quality and quantitation. It is possible that two sets of reconstruction parameters may provide optimal results in a clinical setting—creating one optimized for lesion detection and another optimized for lesion quantitation.

## Conclusions

This study evaluated quantitation of PSMA PET using the anthropomorphic Probe-IQ phantom embedded with radioactive epoxy spheres. BSREM with *β* = 200–400 and OSEM with 2–5 iterations resulted in the most accurate and robust measurements of SUV_mean_, MTV, and TTU for imaging conditions in [^18^F] PSMA PET/CT images. Based on our results, SUV_max_ is not recommended for PSMA PET due to its lack of precision and dependence on the image reconstruction parameters. Differences resulting from reconstruction parameters can be minimized by using SUV_peak_ or SUV_apex_, particularly for small, high-contrast lesions that are characteristic of PSMA scans. When computing metabolic tumour volume, gradient segmentation is preferred over 40% fixed thresholding because it was more robust for different lesion sizes and reconstruction parameters. This study is relevant to clinical trials that aim to reduce variability and improve harmonization of PSMA PET imaging studies.

## Supplementary Information

**Additional file 1.** Supplemental tables and figures for phantom study and patient analysis.

## Data Availability

The datasets used and/or analysed during the current study are available from the corresponding author on reasonable request.

## Abbreviations

BSREM: Block sequential regularized expectation maximization
CNR: Contrast-to-noise ratio
MAE: Mean absolute error
MIM: MIM Software Inc.
MTV: Metabolic tumour volume
OSEM: Ordered subsets expectation maximization
PET: Positron emission tomography
PSMA: Prostate specific membrane antigen
RC: Recovery coefficient
ROI: Region-of-interest
SNR: Signal-to-noise ratio
SUV: Standardized uptake value
TTU: Total tumour uptake
^22^Na: Sodium-22
[^18^F]FDG: [^18^F]fluorodeoxyglucose
40% FT: 40% of SUV_max_ fixed threshold
