# Supplementary material for: Quantitative evaluation of PSMA PET imaging using a realistic anthropomorphic phantom and shell-less radioactive epoxy lesions
Source: EJNMMI Phys. 2022 Jan 15;9:2. doi: 10.1186/s40658-021-00429-9 (PMC8761183; doi:10.1186/s40658-021-00429-9)
Supplement: Supplementary file 1 — Additional file 1. Supplemental tables and figures for phantom study and patient analysis. [file 40658_2021_429_MOESM1_ESM.docx]

**Supplement**

**Supplemental Tables:**

**Supplemental Table 1:** Median, 1^st^ and 3^rd^ quartile [Q1,Q3] for contrast and contrast-to-noise ratios are reported as $Median [Q1\text{-}Q3]$, for images reconstructed using OSEM. [^18^F]DCFPyL included 37 lesions manually segmented from 33 prostate cancer patients. Spheres (4-16mm) were measured for epoxy infused with 28.8 kBq/mL and 57.6 kBq/mL [^22^Na]NaCl.

|  | Contrast_mean_ (kBq/mL) | Contrast_max_ (kBq/mL) | CNR_mean_ | CNR_max_ |
| --- | --- | --- | --- | --- |
| **[^18^F]DCFPyL** | 7.15 [5.5-17.1] | 10.41 [7.7-28.6] | 26.0 [17.4-60.0] | 41.2 [29.6-102.5] |
| **^22^Na Spheres (57.6 kBq/mL)** | 4.47 [3.0-6.0] | 6.07 [4.2-8.0] | 31.2 [14.9-57.3] | 48.2 [22.4-76.2] |
| **^22^Na Spheres (28.8 kBq/mL)** | 5.61 [4.8-6.0] | 6.64 [5.6-6.8] | 32.7 [25.3-51.5] | 40.2 [27.7-60.4] |

**Supplemental Table 2:** Linear fits comparing difference in measured and true value, plotted versus true value for metabolic tumour volume (MTV) and total lesion glycolysis (TTU).

|  | **OSEM (24 subsets)** | **OSEM (32 subsets)** | **BSREM (32 subsets)** |
| --- | --- | --- | --- |
| TMTV (40% FT) | y= -1.49x+1.40  R^2^=0.115 | y= -1.25x+0.976  R^2^= 0.141 | y= -3.17x+4.47  R^2^= 0.158 |
| TMTV (gradient) | y= -0.882x+0.163  R^2^= 0.995 | y= -0.892x+0.162  R^2^= 0.996 | y= -0.815x+0.141  R^2^= 0.987 |
| TTU (40% FT) | y= -0.540x+0.141  R^2^= 0.4708 | y= -0.504x+4.17  R^2^= 0.527 | y= -0.649x+13.3  R^2^= 0.345 |
| TTU (gradient) | y= -0.698x+1.07  R^2^= 0.982 | y= -0.697x+1.46  R^2^= 0.982 | y= -0.685x+0.192  R^2^= 0.979 |

**Supplemental Table 3:** Mean values and 1^st^ / 3^rd^ quartile (shown in square brackets) for the measured lesions in the [^18^F]DCFPyL scan reconstructed with OSEM and BSREM algorithms. SUV, MTV, and TTU listed with units kBq/mL, mL, and kBq, respectively.

| **Metric** | **OSEM (24 subsets)** | **OSEM (32 subsets)** | **BSREM (32 subsets)** |
| --- | --- | --- | --- |
| SUV_max_ | 42.7 [12.3 – 84.4] | 48.0 [14.0 – 95.5] | 36.3 [9.8 – 60.0] |
| SUV_peak_ | 13.5 [5.9 – 19.6] | 14.2 [6.7 – 21.6] | 13.0 [5.4 – 20.8] |
| SUV_apex_ | 22.4 [8.5 – 39.9] | 24.3 [9.6 – 43.7] | 20.9 [7.4 – 39.2] |
| SUV_mean_ (40% FT) | 28.3 [6.9 – 57.4] | 32.9 [8.8 – 64.7] | 24.3 [5.6 – 40.1] |
| SUV_mean_ (gradient) | 26.6 [9.1 – 48.1] | 28.7 [10.5 – 50.8] | 23.8 [8.3 – 42.6] |
| MTV (40% FT) | 0.88 [0.38 – 1.09] | 0.68 [0.32 – 0.66] | 1.86 [0.29 – 3.67] |
| MTV (gradient) | 0.24 [0.20 – 0.26] | 0.28 [0.25 – 0.32] | 0.29 [0.21 – 0.34] |
| TTU (40% FT) | 12.8 [6.3 – 14.7] | 12.2 [5.8 – 16.1] | 16.9 [10.4 – 21.5] |
| TTU (gradient) | 7.3 [2.0 – 10.9] | 8.5 [2.7 – 13.5] | 7.4 [2.1 – 13.2] |

**Supplemental Figures:**


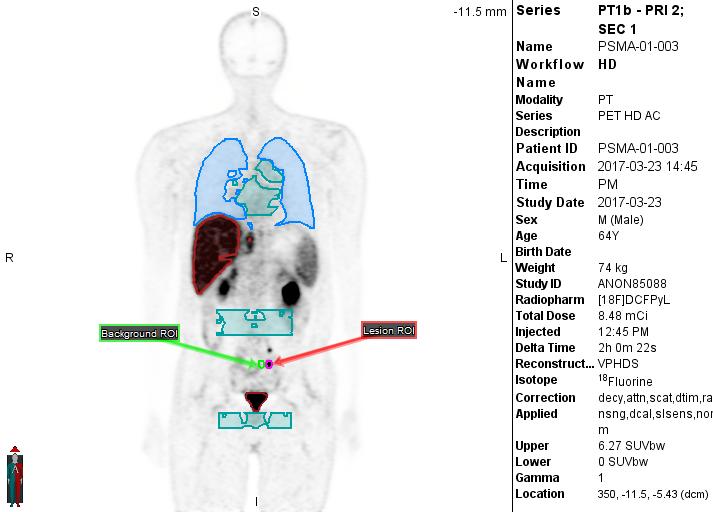


**Supplemental Figure 1:** Lesion and organ ROI segmentations (MIM Software) of [^18^F]DCFPyL PET patient.

af


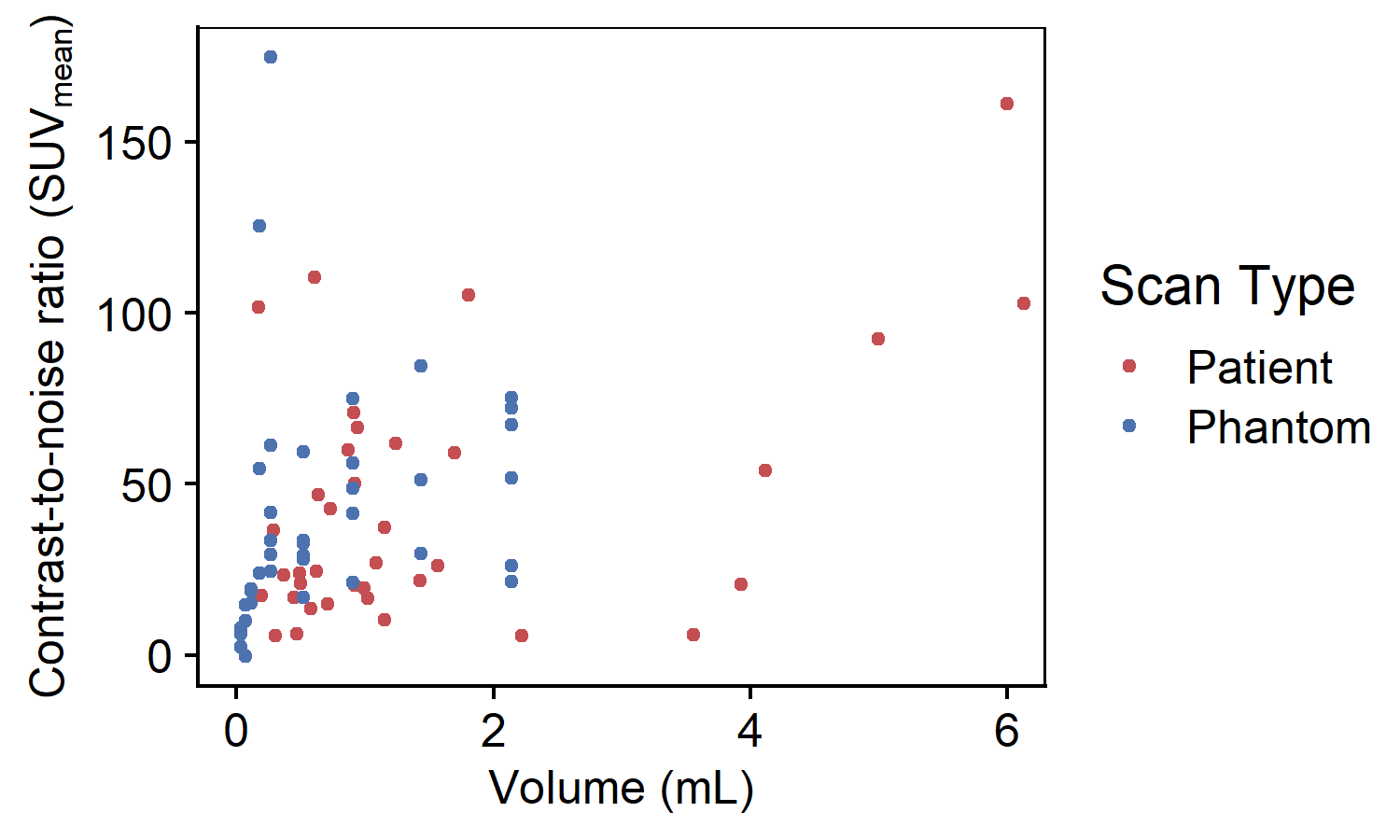


**Supplemental Figure 2:** SUV_mean_ contrast-to-noise ratio vs. volume for OSEM reconstructed images. (Red) 37 lesions manually segmented from 33 prostate cancer patients scanned with ^18^F-DCFPyL. Median [Q1 – Q3] of CNR was 26.0 [17.4 – 60.0], while volume was 0.91mL [0.61 – 1.58]. (Blue) 39 PET Edge+ segmentations of 57.4 kBq/mL lesions with background resembling [^18^F]DCFPyL (1.8 kBq/mL).


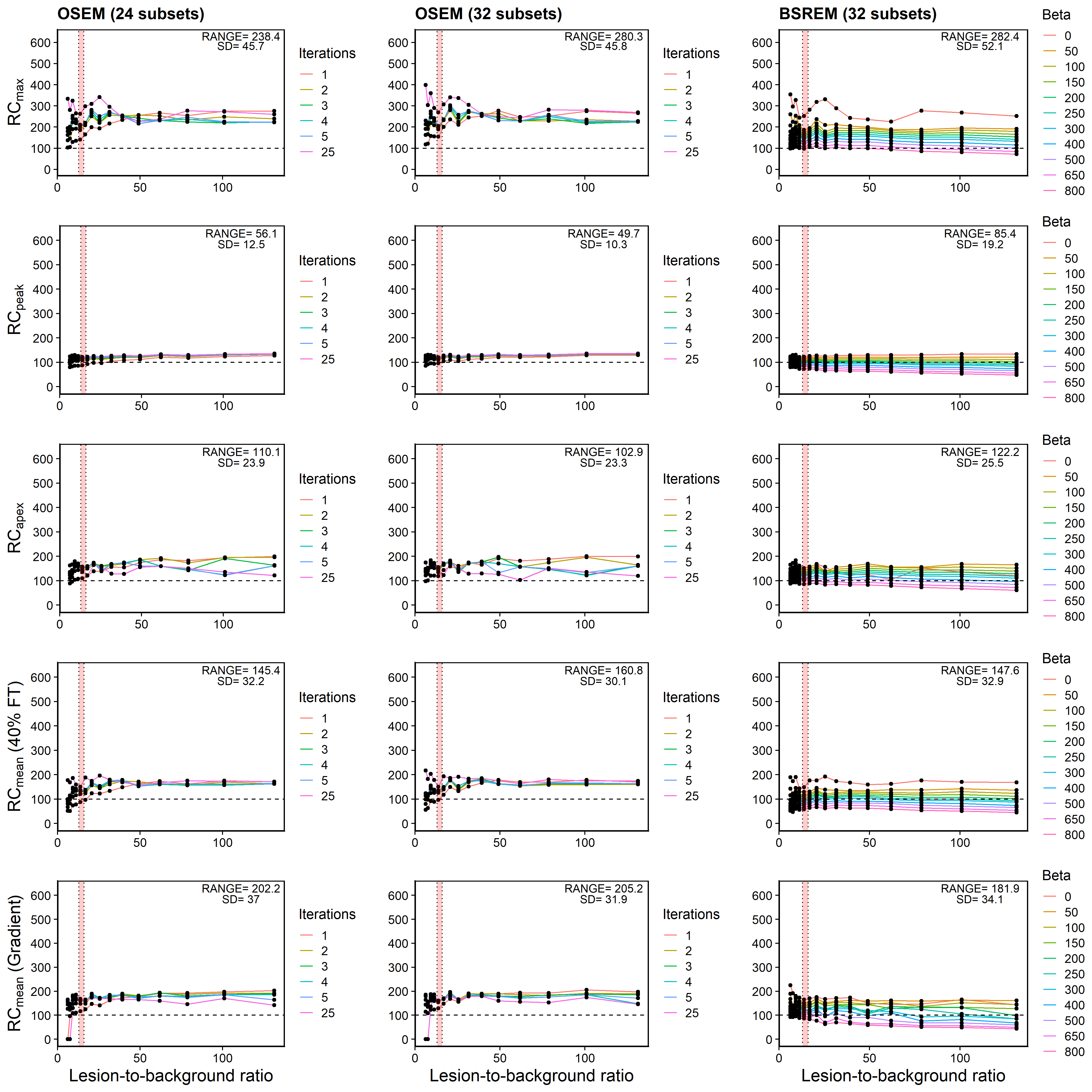


**Supplemental Figure 3:** Recovery concentration coefficient versus lesion-to-background ratio for 16mm lesion measured in Probe-IQ pelvis. (Top to bottom) Max, Peak, Apex, and Mean (40% FT and gradient). (Left to right) Reconstruction algorithms using OSEM+PSF (24 and 32 subsets respectively) and BSREM. Range and standard deviation of recovery coefficients annotated on plots. [^18^F]DCFPyL background activity levels represented by red shaded region.


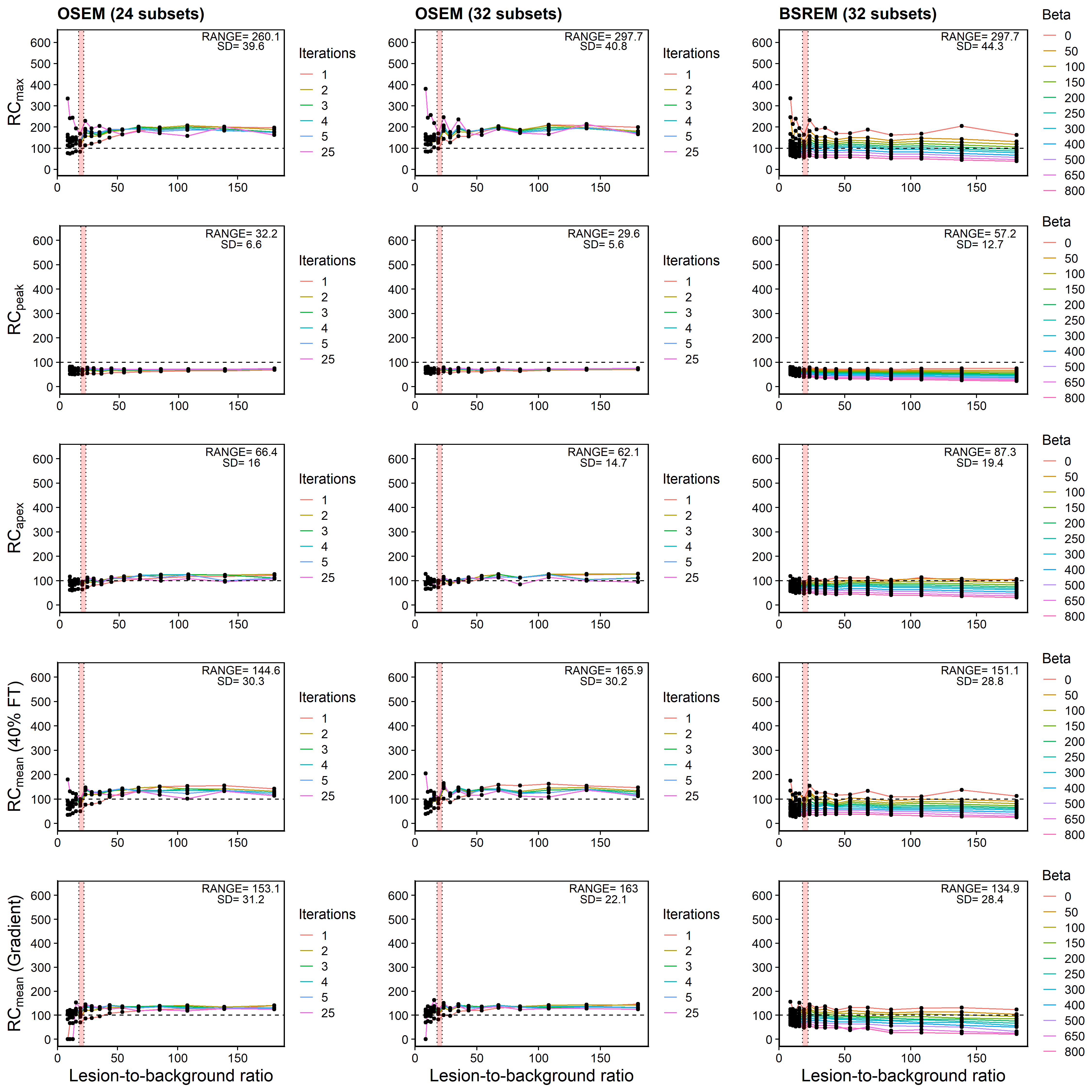


**Supplemental Figure 4:** Recovery concentration coefficient versus lesion-to-background ratio for 12mm lesion measured in Probe-IQ pelvis. (Top to bottom) Max, Peak, Apex, and Mean (40% FT and gradient). (Left to right) Reconstruction algorithms using OSEM+PSF (24 and 32 subsets respectively) and BSREM. Range and standard deviation of recovery coefficients annotated on plots. [^18^F]DCFPyL background activity levels represented by red shaded region.


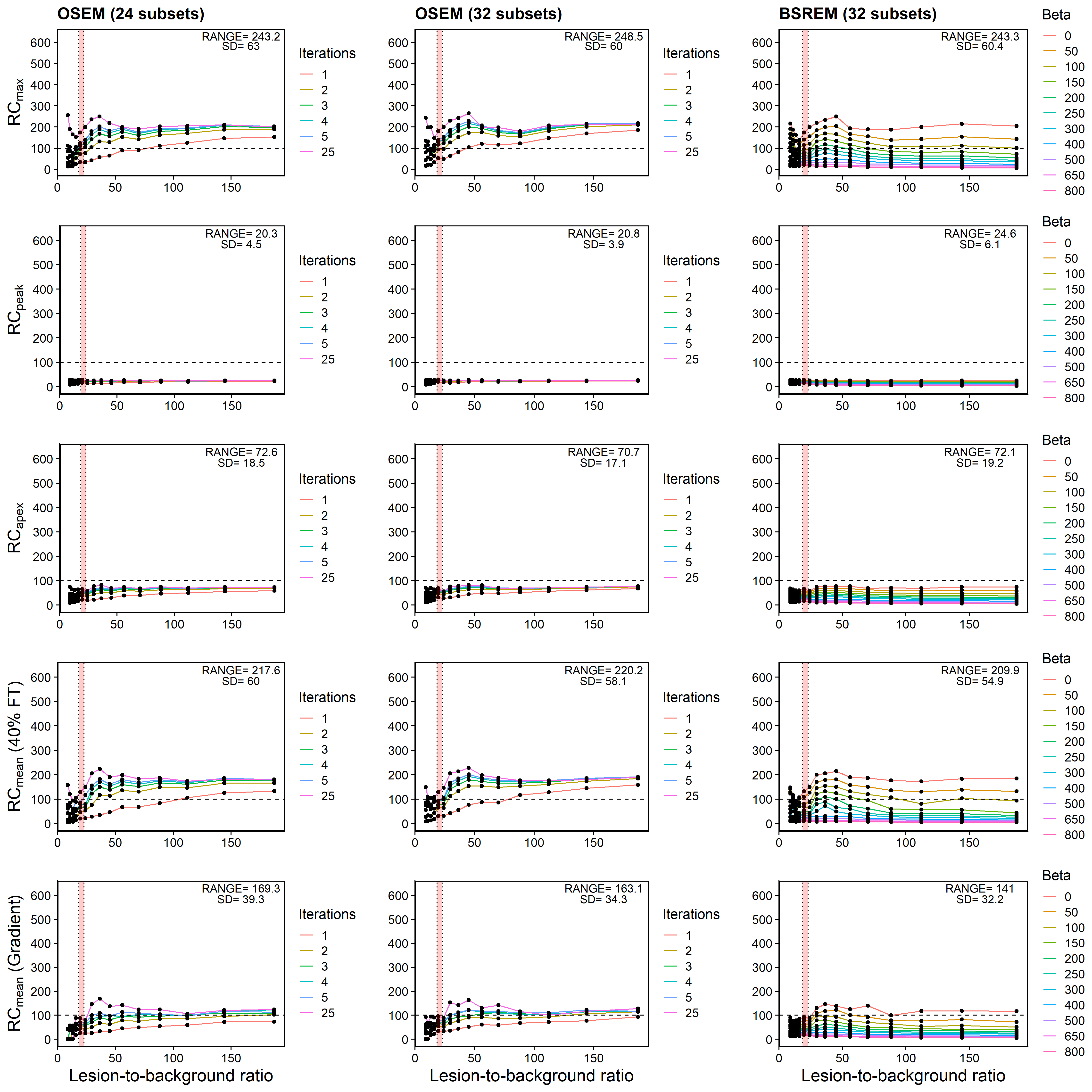
**Supplemental Figure 5:** Recovery concentration coefficient versus lesion-to-background ratio for 8mm lesion measured in Probe-IQ pelvis. (Top to bottom) Max, Peak, Apex, and Mean (40% FT and gradient). (Left to right) Reconstruction algorithms using OSEM+PSF (24 and 32 subsets respectively) and BSREM. Range and standard deviation of recovery coefficients annotated on plots. [^18^F]DCFPyL background activity levels represented by red shaded region.


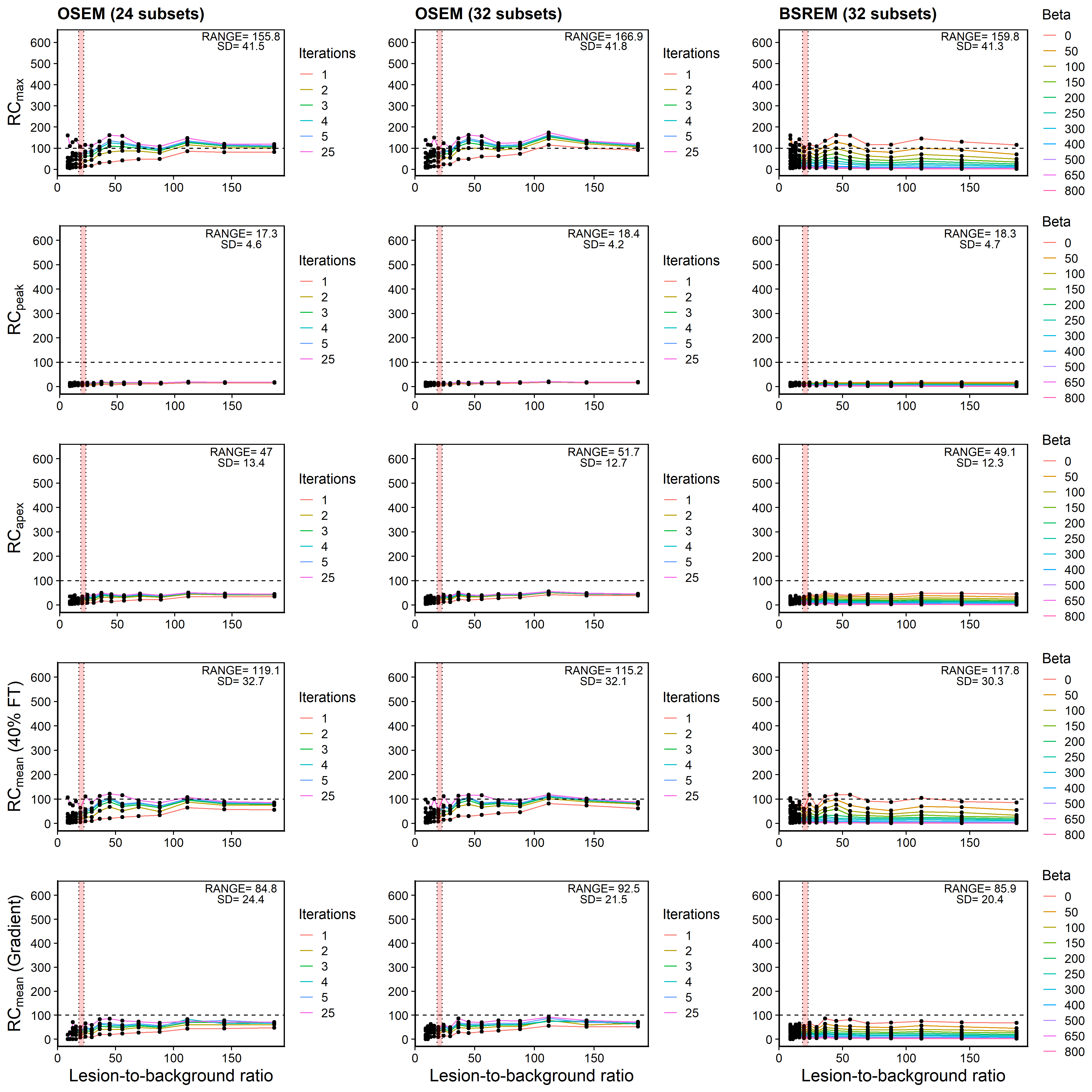


**Supplemental Figure 6:** Recovery concentration coefficient versus lesion-to-background ratio for 7mm lesion measured in Probe-IQ pelvis. (Top to bottom) Max, Peak, Apex, and Mean (40% FT and gradient). (Left to right) Reconstruction algorithms using OSEM+PSF (24 and 32 subsets respectively) and BSREM. Range and standard deviation of recovery coefficients annotated on plots. [^18^F]DCFPyL background activity levels represented by red shaded region.


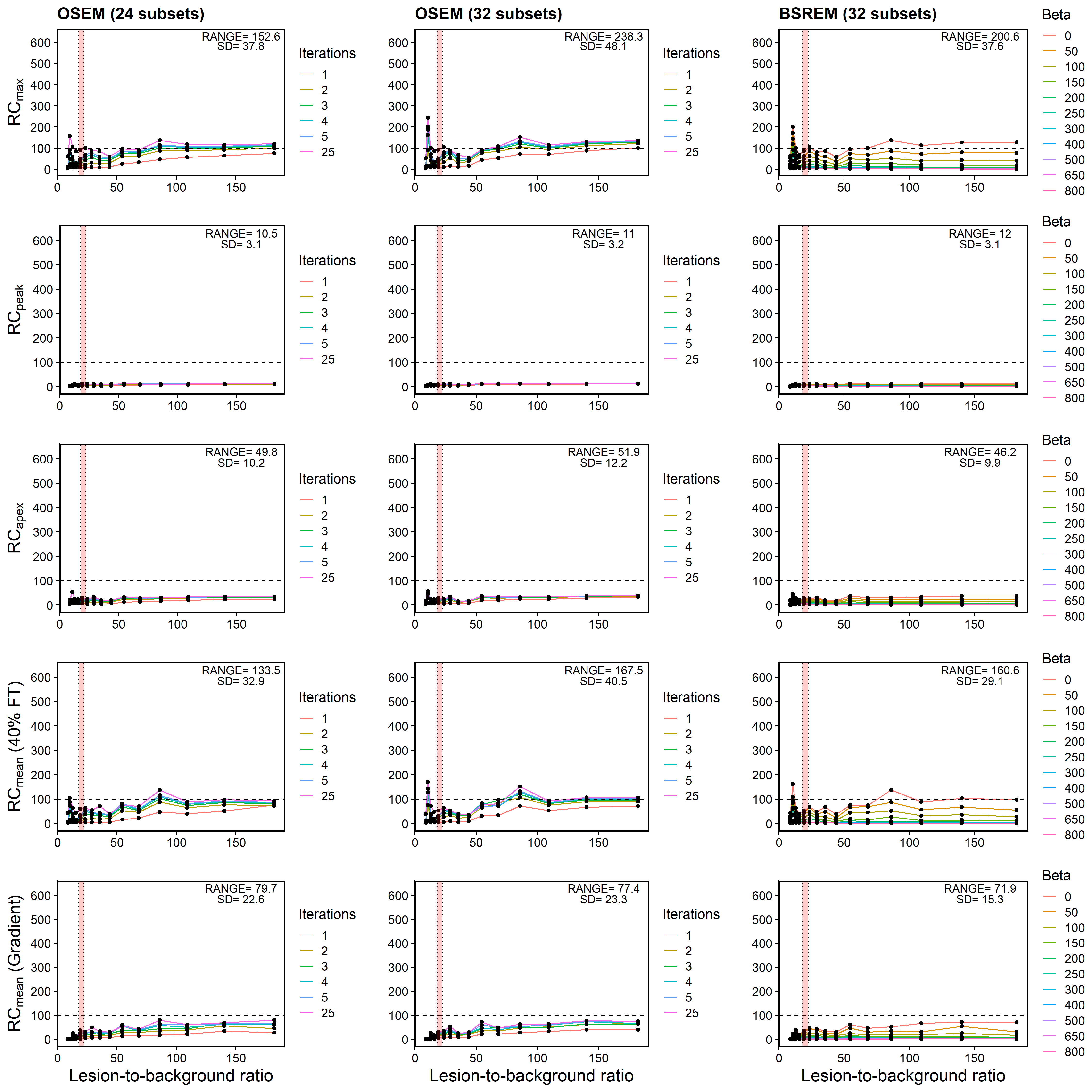


**Supplemental Figure 7:** Recovery concentration coefficient versus lesion-to-background ratio for 6mm lesion measured in Probe-IQ pelvis. (Top to bottom) Max, Peak, Apex, and Mean (40% FT and gradient). (Left to right) Reconstruction algorithms using OSEM+PSF (24 and 32 subsets respectively) and BSREM. Range and standard deviation of recovery coefficients annotated on plots. [^18^F]DCFPyL background activity levels represented by red shaded region.


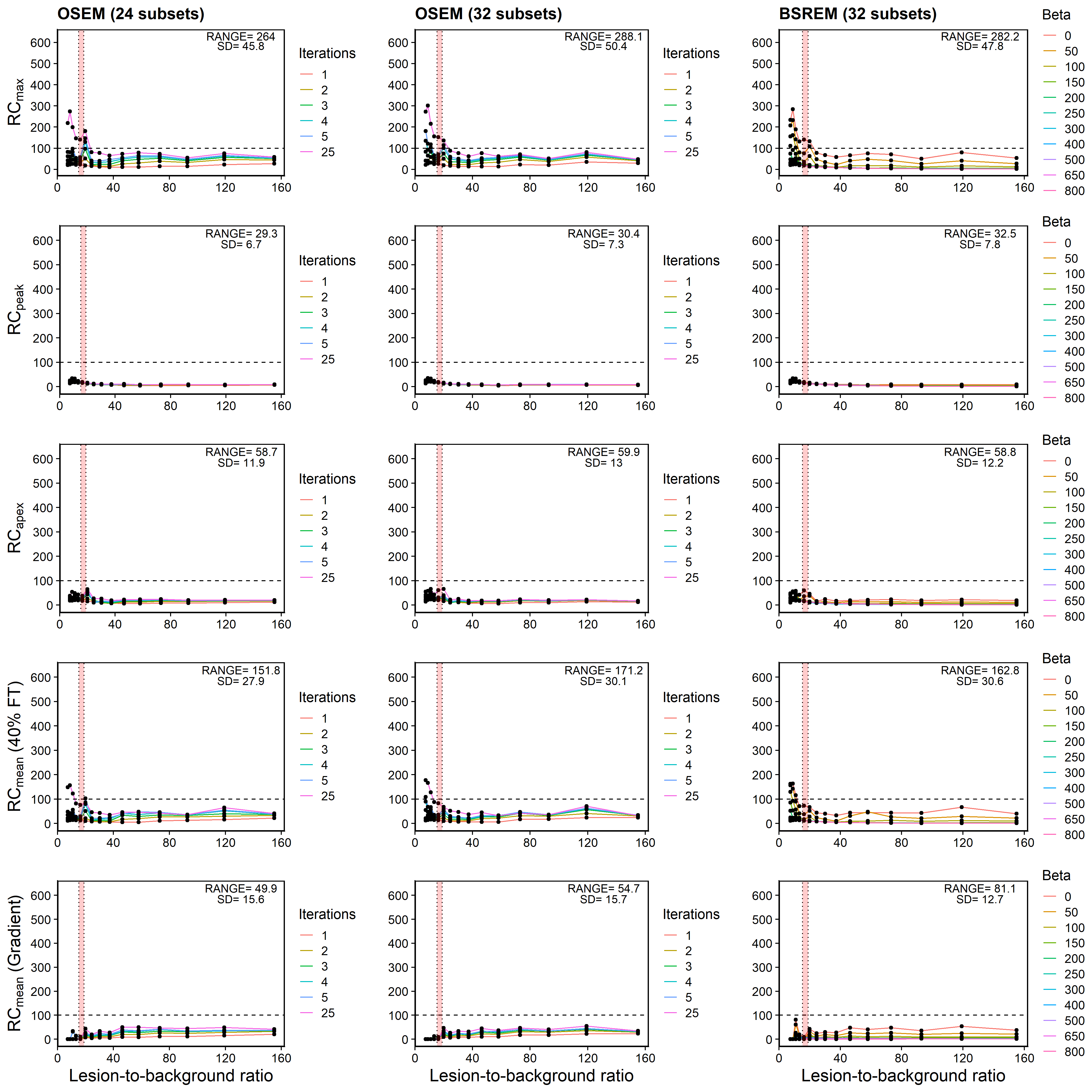


**Supplemental Figure 8:** Recovery concentration coefficient versus lesion-to-background ratio for 5mm lesion measured in Probe-IQ pelvis. (Top to bottom) Max, Peak, Apex, and Mean (40% FT and gradient). (Left to right) Reconstruction algorithms using OSEM+PSF (24 and 32 subsets respectively) and BSREM. Range and standard deviation of recovery coefficients annotated on plots. [^18^F]DCFPyL background activity levels represented by red shaded region.


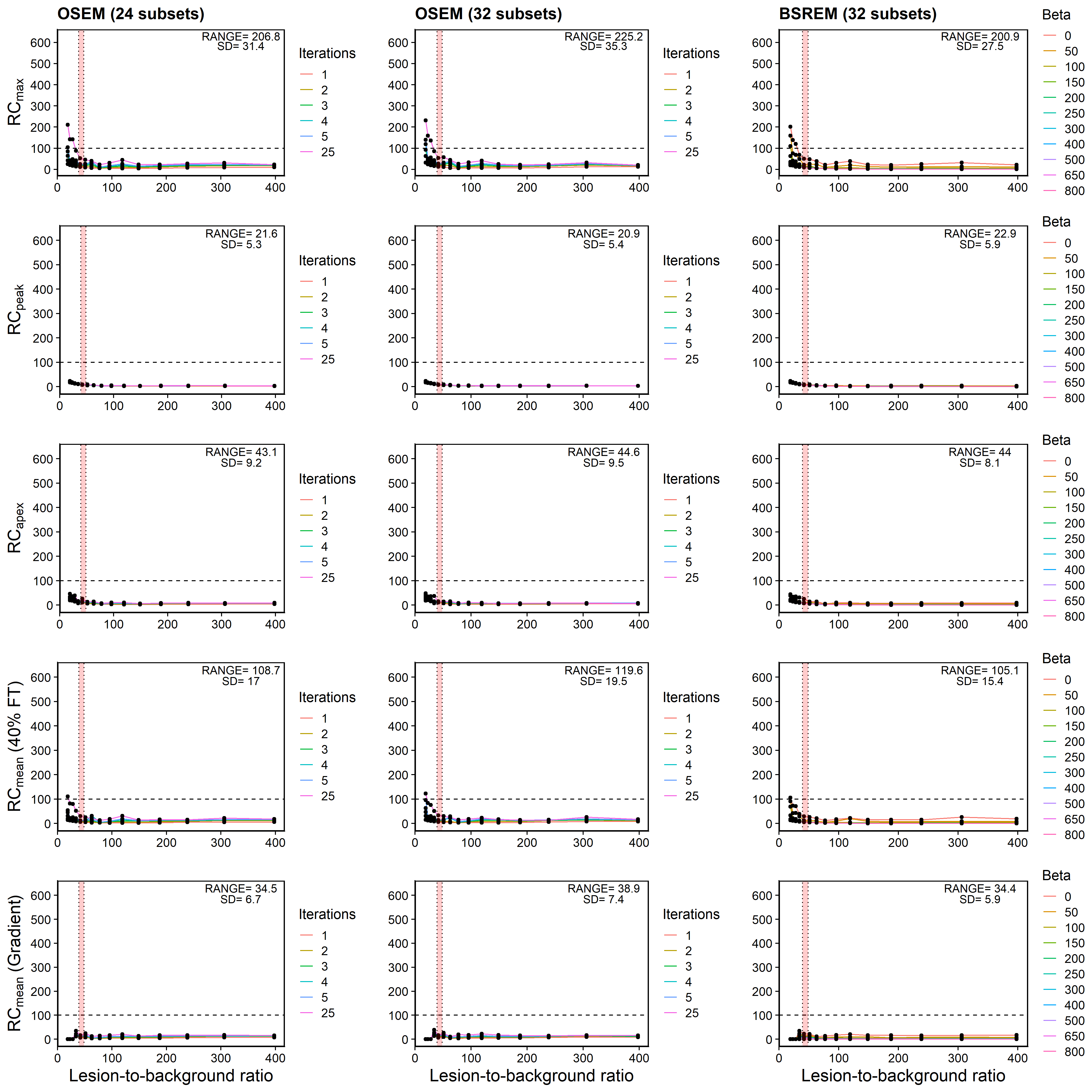
**Supplemental Figure 9:** Recovery concentration coefficient versus lesion-to-background ratio for 4mm lesion measured in Probe-IQ pelvis. (Top to bottom) Max, Peak, Apex, and Mean (40% FT and gradient). (Left to right) Reconstruction algorithms using OSEM+PSF (24 and 32 subsets respectively) and BSREM. Range and standard deviation of recovery coefficients annotated on plots. [^18^F]DCFPyL background activity levels represented by red shaded region.


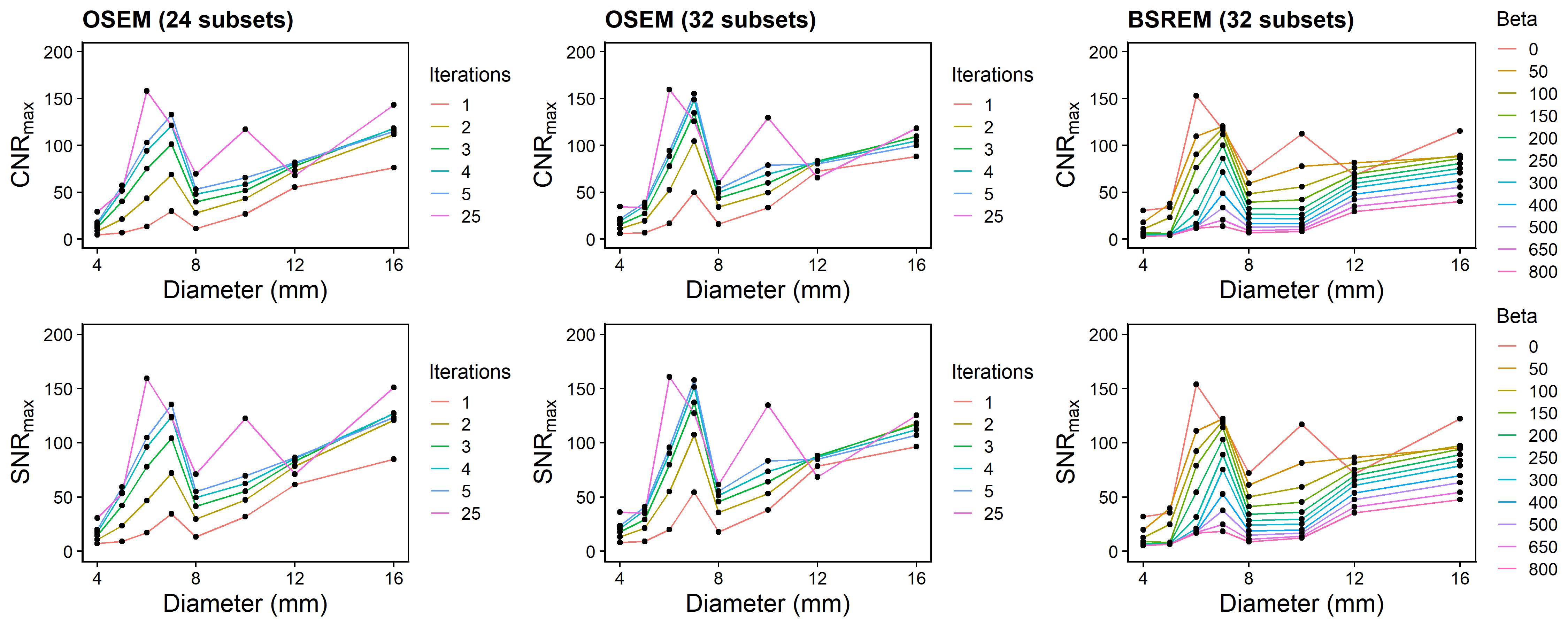


**Supplemental Figure 10:** (Top to bottom) Contrast-to-noise ratio and signal-to-noise ratio vs. lesion diameter using RC_max_ metric. (Left to right) Reconstruction algorithms using OSEM+PSF (24 and 32 subsets respectively) and BSREM.


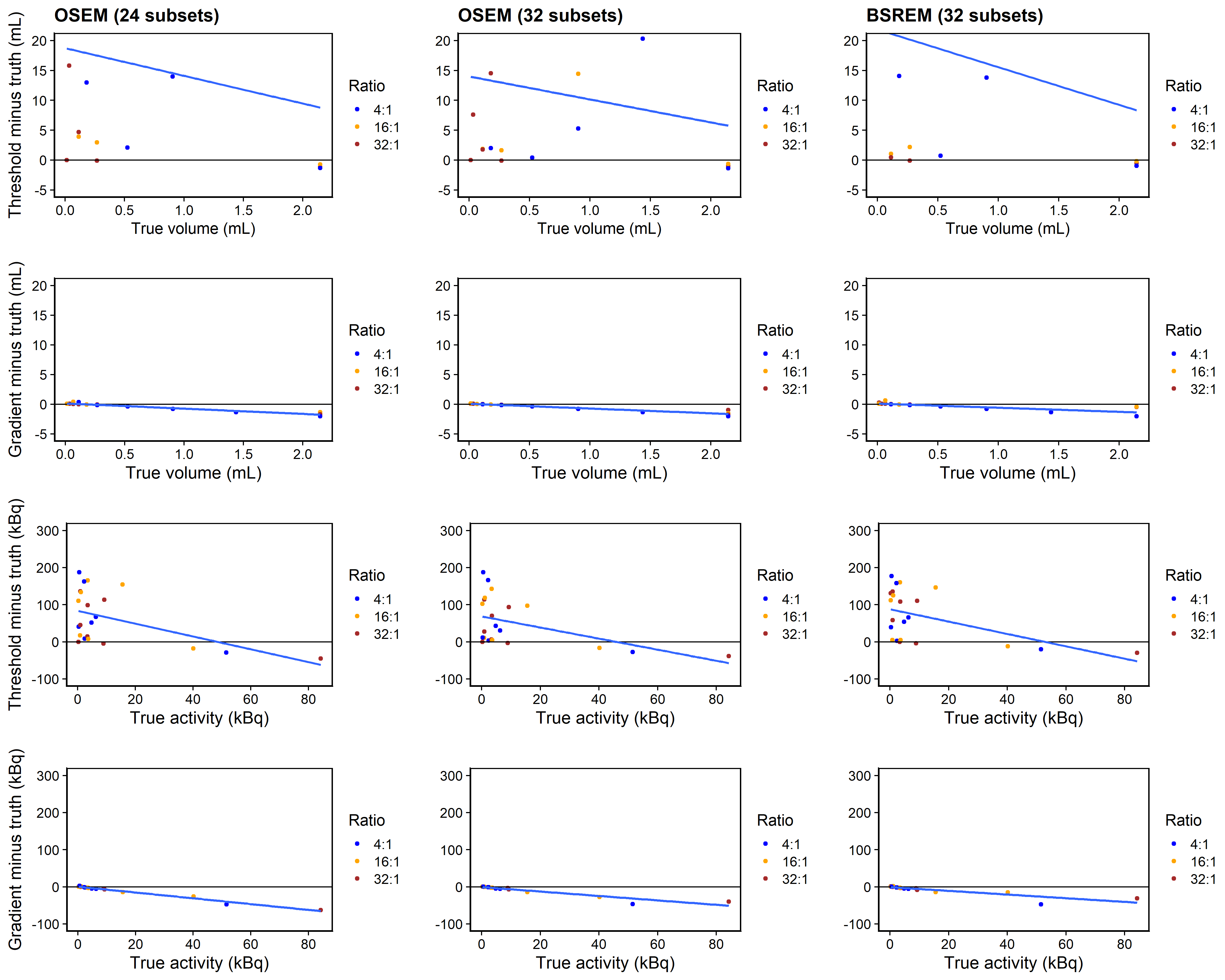


**Supplemental Figure 11:** Difference between segmentation and truth, plotted vs. truth for metabolic tumour volume (MTV) and total tumour uptake (TTU) in the anthropomorphic phantom. (Top to bottom) 40% SUV_max_ fixed threshold and gradient-based methods. (Left to right) Reconstruction algorithms using OSEM+PSF (24 and 32 subsets respectively) and BSREM. Blue line indicates overall fit.
